# The Masticatory Activity Interference in Quantitative Estimation of CA1, CA3 and Dentate Gyrus Hippocampal Astrocytes of Aged Murine Models and under Environmental Stimulation

**DOI:** 10.3390/ijms24076529

**Published:** 2023-03-31

**Authors:** Marília da Cunha Feio Leal, Fabio Leite do Amaral Junior, Bernardo Freire da Silva Arruda, Juliana Ayumi Azevedo Kurosawa, Amanda Almeida Vieira, Júlia Corrêa Campos Maia, Viviana Virgínia Bezerra Scalfoni, Antonio Morais da Silveira Junior, Matheus Oliveira Feijó, Fernanda Beatriz Araújo de Albuquerque, Maria Helena Moutinho Marta, Marina Paula Nobre Normando, Alana Gabriele Oliveira Cabeça da Silva, Fernanda Catharina Pires da Trindade, Fabíola de Carvalho Chaves de Siqueira Mendes, Marcia Consentino Kronka Sosthenes

**Affiliations:** 1 Laboratório de Investigações em Neurodegeneração e Infecção, Instituto de Ciências Biológicas, Hospital Universitário João de Barros Barreto, Universidade Federal do Pará, Belém 66073-005, PA, Brazil; 2 Curso de Medicina, Centro Universitário do Estado do Pará, Belém 66613-903, PA, Brazil

**Keywords:** masticatory activity, neurodegeneration, optical fractionator, CA1, CA3, dentate gyrus, astrocytes

## Abstract

Studies indicating the influence of masticatory dysfunction, due to a soft diet or lack of molars, on impairing spatial memory and learning have led to research about neuronal connections between areas and cell populations possibly affected. In this sense, with scarce detailed data on the subfields of hippocampus in dementia neurodegeneration, there is no information about astrocytic responses in its different layers. Thus, considering this context, the present study evaluated the effects of deprivation and rehabilitation of masticatory activity, aging, and environmental enrichment on the stereological quantification of hippocampal astrocytes from layers CA1, CA3, and DG. For this purpose, we examined mature (6-month-old; 6M), and aged (18-month-old; 18M) mice, subjected to distinct masticatory regimens and environments. Three different regimens of masticatory activity were applied: continuous normal mastication with hard pellets (HD); normal mastication followed by deprived mastication with equal periods of pellets followed by soft powder (HD/SD); or rehabilitated masticatory activity with equal periods of HD, followed by powder, followed by pellets (HD/SD/HD). Under each specific regimen, half of the animals were raised in standard cages (impoverished environment (IE)) and the other half in enriched cages (enriched environment (EE)), mimicking sedentary or active lifestyles. Microscopic stereological, systematic, and random sampling approaches with an optical dissector of GFAP-immunolabeled astrocytes were done, allowing for an astrocyte numerical estimate. Stratum moleculare and hilus, from the dentate gyrus (DG) and Strata Lacunosum-Moleculare, Oriens, and Radiatum, similarly to the dentate gyrus, showed no significant change in any of the investigated variables (age, diet, or environment) in these layers. However, in Stratum radiatum, it was possible to observe significant differences associated with diet regimens and age. Therefore, diet-related differences were found when the HD 18M IE group was compared to the HD/SD/HD 18-month-old group in the same environment (IE) (*p* = 0.007). In the present study, we present modulatory factors (masticatory function, environmental enrichment, and aging) for the differentiated quantitative laminar response in the hippocampal regions, suggesting other studies to read the plasticity and responsiveness of astrocytes, including the molecular background.

## 1. Introduction

The interaction between chewing and cognitive impairment has been the subject of intense investigation in recent years, with masticatory activity being pointed out as a factor in the preservation of brain cognitive functions [1]. Neural circuits connecting oral cavity structures to the Central Nervous System (CNS) transmit proprioceptive information during mastication and reach the hippocampus via the thalamus and cerebral cortex [2,3]. The coordinated muscles contraction during masticatory activity dictates the timing and activation of motoneurons in the brainstem and spinal cord, which are governed by neural networks associated with central pattern generators (CPG) [4,5].

Tooth loss as an agent for reducing masticatory capacity has conditioned a greater risk of cognitive impairment [6,7,8,9,10,11], where restoration of mastication by rehabilitative means would have a significant role in preventing and improving cognitive functions even in patients with neurodegenerative disease [12]. Although most studies show a positive association between chewing and cognitive function [7,13], the translational statement remains to be better established [14], as well as the elucidation of divergent results [9,15].

Experimentally, masticatory impairment, which interferes with learning and memory performances, has led to tracking morphological and cellular changes [1,16,17,18,19,20]. The masticatory deficit was able to decrease the amount of hippocampal pyramidal cells in CA1 and CA3 (Corno Ammonis) [21], in addition to reducing brain blood and oxygen supply [19,22,23], increasing plasma corticosterone levels [24], reducing BDNF (Brain-derived neurotrophic factor), synaptic activity, neurogenesis in the dentate gyrus (DG), and gray matter volume in the temporal lobe [25]. Furthermore, the number of astrocytes in the hippocampus of experimental animal models has been shown to be susceptible to masticatory disorders [20]. Taslima et al. (2021) [26] showed that tooth extraction in AD model mice resulted in an increase in the number of hippocampal astrocytes. Astrocytic changes linked to morphological complexity in the DG molecular layer and changes in laminar distribution in CA1 were also observed, mainly when reduced activity was associated with aging and a depleted environment [17,27]. A recent study showed that posterior tooth loss also induced astrogliosis in the hippocampus and hypothalamus of aged mice compared to young ones [24]. On the other hand, more recent evidence has strengthened the hypothesis that brain aging alone is not accompanied by significant changes in the number of astrocytes [25]. Further investigations about the interaction between masticatory activity and aging are necessary in order to elucidate the astrocytic behavior in response to changes in these variables.

Under physiological conditions, astrocytes influence cognitive function by providing support to neurons, regulating the neurotransmitter system, processing synaptic information, transmission, and plasticity, which are basic functions for information processing and memory consolidation [28,29,30,31]. However, under pathological conditions or during aging, they exhibit quantitative and functional changes due to the loss of their original characteristics [32]. Cell-specific transcriptomics evidenced massive changes in gene expression in astrocytes when they became reactive [32]. Furthermore, reactive astrocytes underwent morphological, molecular, and functional changes in response to injury, disease, or infection of the CNS, using functional parameters to define and potentiate their neuro- and glioprotective actions claims to be hallmarked [33,34].

Despite the changes associated with the senescent brain, studies indicate that maintenance of masticatory activity and environmental enrichment are factors that could minimize the impact of age on cognitive impairment [18,35,36,37,38,39,40,41]. Depending on characteristics of environment and lifestyle, plastic changes in the brain can be strengthened or degraded [42]. Current literature emphasizes that an enriched environment and the preservation of mastication are conditions that can modulate hippocampal aging and neurodegeneration, producing significant changes at behavioral, cellular, and molecular levels [35,36,37,38,39,40]. A recent study observed that long-term environmental enrichment was able to significantly increase the number of resident astrocytes in the dentate gyrus and in CA3 [43]. However, the real extent of quantitative astrocytic alterations related to these variables has been poorly investigated, and this is the first study that analyzes such a broad numerical estimate of these conditions.

Although there are scarce data from hippocampal subfields in demential neurodegeneration, revealing neuronal loss and astrogliosis, especially in the CA1 region [44], there is no detailed information about astrocytic responses in its different layers, or even in CA3 and DG. Thus, considering this context, the present study seeks to evaluate the effects of deprivation and rehabilitation of masticatory activity, aging, and environmental enrichment on stereological quantification using the optical fractionator of hippocampal astrocytes from CA1, CA3, and DG layers, as well as the interaction between these variables.

## 2. Results

Using an optical fractionator to calculate the astrocytic population of DG, CA3, and CA1 subfield layers, the following graphical representations show the results of numerical estimates under the effects of masticatory function, aging, and environmental stimulation.

**Figure 1:**
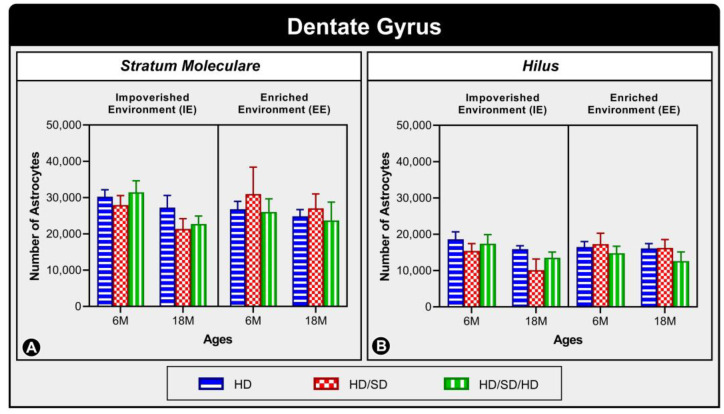
Graphical representation of astrocyte numerical estimation in the dentate gyrus (DG) under the influence of environment, age, and diet regimen. The graph compares the amount of astrocytes present in S. Moleculare (**A**) and Hilus (**B**) of DG in the 6-month-old age group (6M) and 18-month-old age group (18M) reared in standard, impoverished environment (IE), and enriched environment (EE), and submitted to one of three types of diet: HD, hard diet (in blue) = pelleted chow, HD/SD, hard diet/soft diet (in red) = pelleted and mash ration; and HD/SD/HD, hard diet/soft diet/hard diet (in green) = pelleted/powdered/pelleted feed. In this graph, no statistically significant differences were found between the investigated variables.

The following graph (Figure 1) shows the astrocytes numerical estimate in the stratum moleculare (Figure 1A) and hilus (Figure 1B) of the dentate gyrus (DG). The presented results showed no statistically significant difference between the experimental groups in these layers regarding diet regimens, age, or environment stimulation or impoverishment (for detailed data, see Supplementary Materials Tables S1–S8).

**Figure 2:**
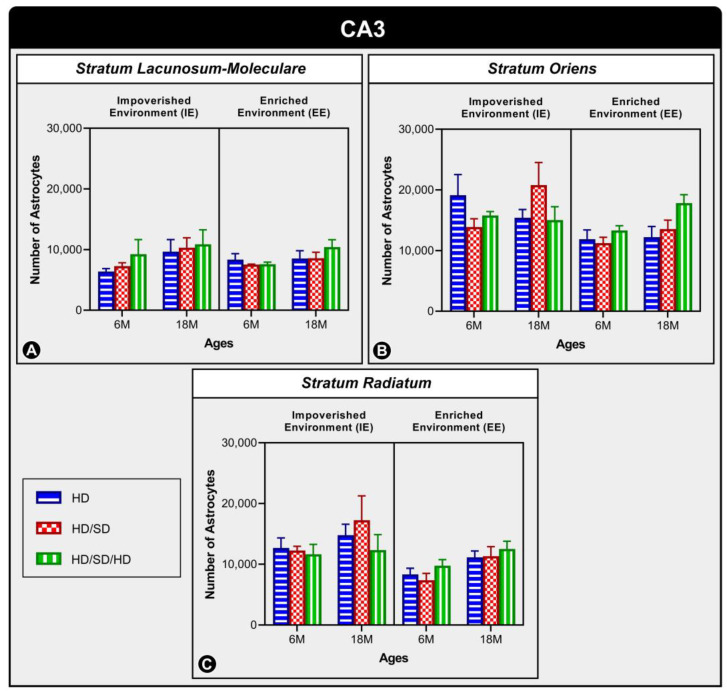
Graphical representation of astrocyte numerical estimation in CA3’s three layers: Stratum Lacunosum-Moleculare (**A**), Stratum Oriens (**B**), and Stratum Radiatum (**C**) under the effects of environment, age, and diet. The graph shows the quantitative results of astrocytes from these layers in the 6-month-old age group (6M) and 18-month-old age group (18M) reared in standard, impoverished environment (IE), and enriched environments (EE) and submitted to one of three types of diet: HD, hard diet (in blue) = pelleted ration; HD/SD, hard diet/soft diet (in red) = pelleted and mashed chow; and HD/SD/HD, hard diet/soft diet/hard diet (in green) = pellet/powdered/pelleted feed. In this graph, no statistically significant differences were found between the investigated variables.

The graph represented at Figure 2, in turn, shows the number of astrocytes in three CA3 layers: Stratum Lacunosum-Moleculare (Figure 2A), Stratum Oriens (Figure 2B), and Stratum Radiatum (Figure 2C), and similarly to the dentate gyrus, it is also observed that there was no significant change in any of the investigated variables (age, diet, or environment) in these layers.

The pyramidal layer was accessed, and cells estimated, but the identified error coefficient constantly exceeded the limit placed as acceptable, requiring the reformulation of its protocol. As the exposed connections could not be influenced by that layer, it was decided to remove them and not include them in the comparisons, requiring later additions.

**Figure 3:**
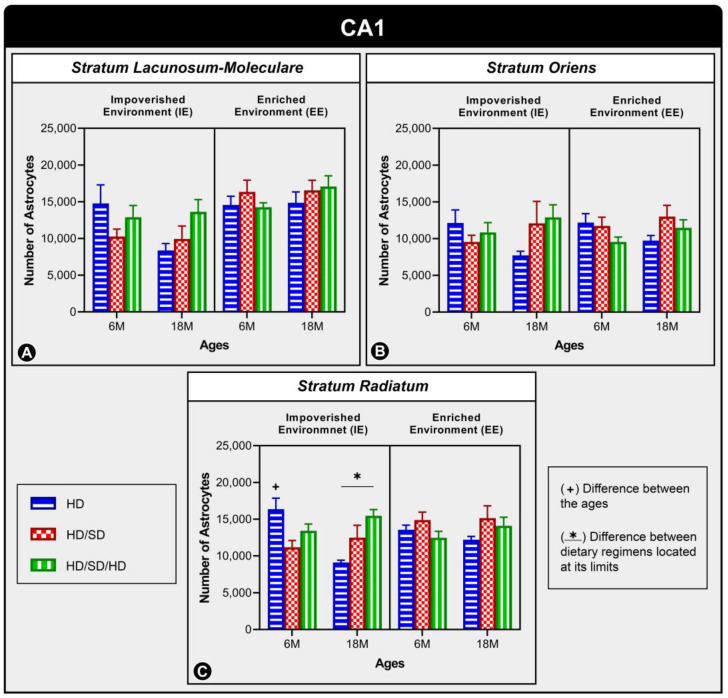
Graphical representation of astrocyte numerical estimation in CA1’s three layers: Stratum Lacunosum-Moleculare (**A**), Stratum Oriens (**B**), and Stratum Radiatum (**C**) under the effects of environment, age, and diet. The graph shows the quantitative results of astrocytes from these layers in the 6-month-old age group (6M) and 18-month-old age group (18M) reared in standard, impoverished environment (IE), and enriched environments (EE) and submitted to one of three types of diet: HD, hard diet (in blue) = pelleted chow, HD/SD, hard diet/soft diet (in red) = pelleted and mashed chow, and HD/SD/HD, hard diet/soft diet/hard diet (in green). In this graph, no statistically significant differences were found between the investigated variables. The symbol (+) indicates a statistically significant age-related difference, while the symbol (*) indicates a difference between diet regimes.

In the CA1 subfield (Figure 3), significant alterations were also not identified for Stratum lacunosum-moleculare (Figure 3A) and Stratum oriens (Figure 3B); however, in Stratum radiatum (Figure 3C), it was possible to observe significant differences associated with age and diet regimens. Therefore, regarding age, there was a difference in the HD group of 6-month-olds (6M) maintained in an impoverished environment (IE) when compared to the HD 18-month-olds created in the same environment (IE) (*p* = 0.001). Diet-related differences were also found when the HD 18M IE group was compared to the HD/SD/HD 18-month-old group in the same environment (IE) (*p* = 0.007).

To assess substantial body weight loss, periodic weigh-ins were performed to monitor the groups. Within the 6M age group, created in IE, the ANOVA-one-way test (*t*-student post-test; *p* < 0.05) revealed that mice subjected to normal (HD) and rehabilitated masticatory activity (HD/SD/HD) showed significantly higher body weights than those mice submitted to reduced masticatory activity (HD/SD group). The same occurred at 18 months of age, when HD weighed significantly higher than the HD/SD and HD/SD/HD diet groups. This increasing condition was also observed in the groups created in EE at 18M age, but no important differences were detected at 6M age (*p* > 0.05).

Thus, as we did not detect robust differences in the quantitative distribution of astrocytes in CA3 and GD and, in the case of CA1, the evident difference was only detected in the Stratum radiatum, it is reasonable to assume that it is not differences related to body weight that would justify the astrocytic changes. Moreover, if Stratum radiatum is analyzed exclusively, we will see that there is no coherence in the results between the astrocytic findings and the animal’s weight. Table 1 shows the mean and standard error values for each experimental group, and Table 2 presents significance values obtained for paired samples.

**Figure 4:**
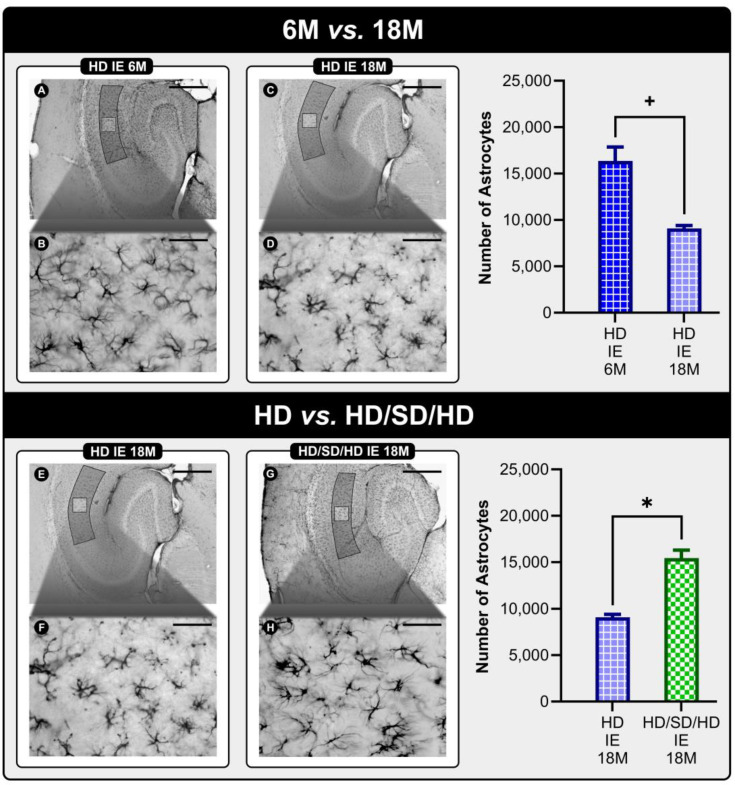
Low-power (4×) (**A**,**C**,**E**,**G**) and medium-power (40×) (**B**,**D**,**F**,**H**) photomicrographs, respectively, of GFAP-immunolabeled sections of experimental groups with significant differences in the number of astrocytes present in the Stratum Radiatum of CA1. When we compare the ages of 6M and 18M of animals housed in the impoverished environment (IE), we see a significant reduction in the total number of astrocytes associated with aging. This reduction is visually identified in photomicrographs (**B**,**D**) due to the lower population density of astrocytes in the latter than in the former photomicrograph. When comparing diet influence on aged animals (18M) submitted to the impoverished environment (IE), masticatory rehabilitation significantly increased the number of astrocytes, making it possible to visually identify this increase in astrocytic population density in the F and H photomicrographs. Scale bars: (**A**,**C**,**E**,**G**) = 250 μm; (**B**,**D**,**F**,**H**) = 25 μm. Abbreviations: HD—hard diet/pelleted feed; SD—soft diet/mashed feed; IE—Impoverished Environment; 6M—6-month-old age group; 18M—18-month-old age group. The symbol (+) indicates a statistically significant age-related difference, while the symbol (*) indicates a difference between diet regimes.

Figure 4 summarizes the results with significant differences. Low-power (4×) and medium-power (40×) photomicrographs illustrate the objects of interest (GFAP-labeled astrocytes) and reveal visually perceptible differences between the populations of astrocytes present in the Stratum radiatum of CA1 between the experimental groups. When we compare the ages of 6M and 18M of animals housed in the impoverished environment (IE), we see a significant reduction in the total number of astrocytes associated with aging. This reduction can be visually identified in photomicrographs B and D due to the lower population density of astrocytes in the latter, HD IE 18M, than in the former photomicrograph, HD IE 6M. When comparing diet influence on aged animals (18M) submitted to the impoverished environment (IE), masticatory rehabilitation significantly increased the number of astrocytes, making it possible to visually identify this increase in astrocytic population density in the F and H photomicrographs, HD IE 18M and HD/SD/HD IE 18M, respectively.

## 3. Discussion

In general, in young animals from the impoverished environment (6M IE), the group with depreciated masticatory activity (HD/SD) shows an astrocytic estimation reduction in relation to the other two groups—under the same conditions of environment and age, HD/SD/HD and HD, when considering CA1. However, in 18M IE, all layers suggest that groups with an altered masticatory activity pattern tend to show a greater number of astrocytes when compared to the control group. In sequence, observing the IE results, we noticed a slight tendency for the HD/SD and HD/SD/HD groups to reveal a slight astrocytosis, with the HD/SD/HD group showing an age-dependent increase. Thus, there is an indication that undergoing changes in masticatory activity, whether its reduction or restoration, are stressful, requiring a greater demand for astrocytes. The stereological estimate identified that the total number of astrocytes and microglial in DG and CA1 differs according to age in C57BL/6J mice, but no references were done individually to hippocampal layers [45]. The study by Cerbai et al. (2012) [46] evaluated the number of astrocytes in the radiatum and pyramidal layers of CA1, and unlike the analysis presented here, used another method for estimation, not using the estimation by the three-dimensional method and optical fractionator. There is also no reference in the literature about an individual survey of strata in CA1, CA3, and DG. Chun et al. (2018) [47] suggested the expectation of a dynamic response of astrocytic morphological and functional properties to environmental stimulation, urging to track the population quantitatively [47]. Thus, there is a suggestion that the proBDNF astrocyte may be a genuine molecular marker of active astrocytes, which are distinct from reactive astrocytes, as these have a hypertrophic profile with aberrant GABA [47].

Regarding the subtle differences revealed in CA1, we identified very similar results among the layers, with Stratum radiatum indicating slightly higher sensitivity. Its location in the vicinity of the pyramidal layer, where Schaffer collateral fibers effect connections in the trisynaptic circuit, as well as the proximity to the subiculum, can expose the astrocytic population to important requirements revealed in an astrocytosis. The oriens layer also indicated a responsive quantitative population of astrocytes, but not of the same intensity, perhaps protected by the compensatory vascularization represented by the hippocampal fissure. The location of place cells—that are considered to be some CA1 and CA3 pyramidal hippocampal neurons responsible for encoding specific locations in an environment—and grid cells—hippocampal neurons activated by a geometric scanning of an environment—may suggest the source of spatial information that would activate cells [48] and subfield layers. This subtle difference was also observed by Cerbai et al. (2012) [46], where the authors observed a decrease in the astrocyte number in the Stratum radiatum in elderly animals. This difference was not observed in the pyramidal layer of old animals or in young animals; the other layers were not evaluated.

In this reasoning and among a range of functions possibly performed by astrocytes, to generate and maintain a microenvironment of adequate support for neuronal performance, the increase in astrocytic quantity may be related to the demand for challenges to neurons, requiring a more responsive profile of astrocytes. Thus, it is reasonable to assume that when faced with high challenges, the reaction should be at the same level, and environmental enrichment could provide support for this feedback. Observed in a synchronous way, spatial memory performances are implemented by sensorimotor and visuospatial stimulation from environmental enrichment and by rehabilitation of masticatory activity, which recovers the learning even in aged groups [39].

The CA1 region is placed as containing vulnerable neurons, while the dentate gyrus contains resistant, durable neurons [49], so much so that when tracking microglia (anti-Iba-1) in animals with alterations in masticatory activity, the area occupied by microglial activity increased considerably in the CA1 region with a peak on the 3rd day and a gradual decline from the 5th day on [49], but not in the control of animals, and as estimates, they were not performed by stereology with the application of the optical fractionator, and it is important to make the association that microglia cannot only respond to neuronal degeneration, but also to their survival [50].

An overview, regardless of the considered layer, suggests that analyzing young animals kept in an environment deprived of stimuli suggesting that astrocytes cannot respond adequately to impact of masticatory activity deprivation, reducing in quantity and being impactful also in their special memory performances, which proved to be the most inefficient compared to other learning groups [17,18,39]. Previously developed studies showed recovery of performance in spatial memory tests when associated with rehabilitation of masticatory activity and environmental enrichment, even in aged animals, bringing them closer to the results of control groups. This situation is not identified in animals kept in a poor stimulation environment or deprived of their masticatory function.

A question of astrocytic maturity can be inferred when in young animals the masticatory activity deprivation is impacting and astrocytes do not react efficiently, in addition to the time length of deprivation being short (3 months, proportionally). That is, age would bring the possibility of maturation to the profile and quality of response of the astrocyte. On the other hand, maintenance in an enriched environment seems to provide favorable conditions for astrocyte action, with a greater number of astrocytes being identified in 6M HD/SD EE when compared to 6M HD/SD IE. In a neuroronal niche, astrocytes and microglia support and interact with neural stem cells (NSCs) through a cell-to-cell mechanism, with contact between astrocytes and NSCs regulating neuronal differentiation in the DG subgranular zone through instructive juxtacrine ephrin-B signaling. [45,51].

In the same way, when we analyze old animals, environmental enrichment seems to give breath to their functions even when considering the HD control group, but with a slight increase in groups involved in changes in chewing activities, either in deprivation or in recovery, perhaps because the situations demand more from astrocytes. To measure from a behavioral and functional point of view, in EE, the 18M HD/SD/HD group presented a good performance, while the 18M HD/SD EE always presents a deficit.

Furthermore, in other studies it was possible to identify that short-term mastication stimuli reduction caused changes in neuronal morphology in the thalamus [52]; however, when considering CA1 astrocytes, a differentiated laminar sensitivity was identified considering masticatory activity deprivation, aging, and environmental enrichment in the present study. In this sense, while it is perceived that there are not such significant differences in the DG and CA3 subfields, those in CA1 are identified, inciting the perception that in evasion of the circuit, the temporo-ammonic pathway does not refer to the regions of CA3 and GD but connects to CA1, making this subfield the direct congruence between the trisynaptic circuit and the temporo-ammonic pathway. Eight-week-old molarless mice did not show altered spatial memory performance parameters or hippocampal astrocyte density; however, extraction associated with zinc deprivation led to an important cognitive decline followed by an increase in astrocyte density in the CA1 region. Although, in adult animals (22-week-olds), regardless of molar extraction or deprivation, no differences were identified in astrocyte density in the CA1 region between groups [53], it is important to observe the activity dysfunction time and the responsive region.

Allied to this, we cannot forget that masticatory function plays an important role in the stomatognathic system and that its central motor control, preservation of hippocampal trophism, and synaptic activity should contribute to the study of neurodegenerative diseases in an innovative and revealing way [21]. Mastication is considered a rhythmic activity like walking and breathing [4], involving muscle contraction orchestrated by networks of motoneurons called central pattern generators (CPG) that properly conduct complex behavior [4,5]. Knowing that most synapses in the CNS occurs in close apposition of astrocytic processes, if neurotransmitters released by neurons adhere to receptors expressed by astrocytes and activate pathways that increase calcium concentration and gliotransmitters that can modulate synaptic transmission, it is reasonable to assume that astrocyte activation impacts on motor control [4,5]. The sensory-motor trigeminal circuit in rats for chewing depends on extracellular regulation of Ca^2+^ by astrocytes [54].

Orosensory stimulation has resulted in a differentiated brain response [55], and in animals it was possible to track the effect of masticatory activity on cognitive function, which tends to decline with advancing age [17,18]. During masticatory activity, orosensory stimuli are received by nerve endings contained in the oral epithelium, dental roots, and muscular bundles, traveling as an ascending pathway to the medulla oblongata, pons, and thalamus to the somatosensory cortex; it is known, however, that there are neuronal connections established with the hippocampus, amygdala, and frontal cortex involving chewing in the performance of memory function [52,56,57]. Previously, a quantitative alteration of thalamic neuron spines was identified as significantly reduced in softened diet groups and accompanied by an upregulation of GABA-related gene expression [52]. In the present study, we present modulatory factors (masticatory function, environmental enrichment, and aging) for the differentiated quantitative laminar response in the hippocampal regions, leaving the need for other studies to read the plasticity and responsiveness of astrocytes, including the molecular background.

In addition to the roles of masticatory activity, aging, and environmental enrichment, circulating gonadal hormones also exert an influence on neuroglial proliferation [58]. The morphology and function of astrocytes, as well as their interaction with other CNS cells, are regulated by sex steroids. Gonadal hormones modulate reactive astrogliosis and reduce the release of pro-inflammatory molecules by astrocytes [59]. In view of hormonal influence on brain tissue homeostasis, sexual dysmorphisms related to the number and morphology of astrocytes have been deeply studied [59]. Such differences have been detected in some regions of the rodent brain, especially the hippocampus. In fact, female animals have a few glial cells that are 25 to 40% higher than their male counterparts [58]. Furthermore, Lei et al. (2003) [60] found that estrogen decreased the amount of microglia and astrocytes in the DG and CA1 of females aged 20–24 months. In the case of the present research, we cannot exclude the effects of serum hormone concentrations on the quantification of astrocytes since we used only female animals. However, given the hypothesis that older females are in conditions of depleted plasma levels of estrogen, we can assume that at least part of the results obtained in older animals may reflect estropause. However, it is also reasonable to assume that the hormonal influence was not relevant to the point of influencing the results obtained, since only in the HD IE group (6M vs. 18M), but not in others, the comparison between ages proved to be significant. Under this analysis, we are led to believe that the results obtained are much more influenced by the other variables studied (masticatory alteration and environment) and do not suffer significant interference from variations in the estrous cycle.

## 4. Materials and Methods

All experimental procedures were fully approved prior to study initiation by the Ethics Committee in Research with Experimental Animals (CEPAE) of the Instituto de Ciências Biológicas from the Universidade Federal do Pará (CEPAE-UFPA: 223-14). The animals were treated in accordance with the guidelines published by the NIH (Guide for the Care and Use of Laboratory Animals).

### 4.1. Animals and Experimental Groups

A total of 60 Swiss albino females (*Mus musculus*) were selected and randomly organized on the 5th postnatal day. Mice remained with their mothers until the 21st postnatal day at a rate of 6 pups per litter. At weaning, experimental groups were organized based on three variables to be investigated: masticatory regimen, age, and environment.

### 4.2. Feeding and Experimental Timeline

Between the 21st postnatal day and the end of the experiment, mice were subjected to one of the three different diet regimens with no distinction in composition or nutritional value. Animals were fed a continuous pellet hard diet (HD) or normal diet, a hard diet followed by a soft diet (HD/SD), or a hard diet followed by a soft diet and again a hard diet (HD/SD/HD).

The period of each diet regimen was defined based on two groups formed from the variable “age: 6-month-old (6M) and 18-month-old (18M)”. The 6M group was composed of mice that completed their experimental time in 6 months and represented young adult mice, while the 18M group was made up of mice that underwent 18 months of study variables and represented aged adult mice.

Alternate diet regimens were equally distributed over time. Mice subjected to the HD/SD diet were fed pellets for the first half of their experimental time and powder diet for the rest of their lifespan, as follows: 6 M mice were fed pellets for 3 months and powder diet for 3 months (3M HD + 3M SD); and 18M mice were fed pellets for 9 months and then a powder diet for 9 months (9M HD + 9M SD).

**Figure 5:**
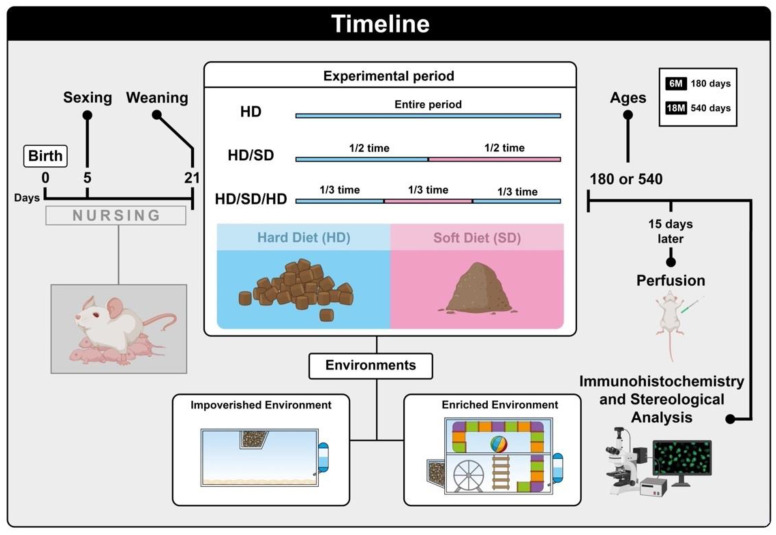
Experimental timeline. Female albino Swiss mice, *Mus musculus*, were selected on the 5th postnatal day and remained breastfeeding until the 21st postnatal day. After this period, the animals were divided into groups that were fed a continuous hard pelleted diet (HD), a hard diet followed by a soft diet (HD/SD group), or a hard diet followed by a soft diet and again a hard diet (HD/SD/HD). The total time of each feeding regimen was 6M (180 days) and 18M (540 days), representing respectively young and aged adult mice. The alternating diets (HD/SD and HD/SD/HD) were equally distributed over the lifespan (1/2 time and 1/3 time). Half of the animals were kept in poor environments in standard laboratory cages (impoverished environment (IE)) and the other half in environmentally enriched cages (enriched environment (EE)). After completing the experimental time for each group, the mice were submitted to transcardiac perfusion, and the brains were removed and submitted to sectioning and immunohistochemical processing for further stereological analysis. Created in BioRender.com.

Likewise, the HD/SD/HD group had its experimental time divided up into 3 periods of equal duration. The first third consisted of a pellet diet followed by a powder diet for the next ^1^/_3_ of the time, followed by a pellet diet again for the final ^1^/_3_ of the experimental time. The 6M mice undergoing a HD/SD/HD diet were fed a pellet diet for 2 months, a powder diet for the next 2 months, and again, a pellet diet for the last 2 months (2M HD + 2M SD + 2M HD). In the same way, 18M mice subjected to HD/SD/HD diet as follow: 6M HD + 6M SD + 6M HD. We assumed that the powder diet required less masticatory activity as compared to the pellet diet. Figure 5 summarizes the experimental procedures.

Figure 5 summarizes the experimental design used in the present study. The chronological order of events is represented spatially, from left to right, so that the diagrams on the left correspond to what was performed earlier during the experimental period and those on the right later. The types of feed (pellets as hard and powdered diet as soft) offered to the animals are illustrated in the figure, as is the temporal division of each of the diet groups (HD, HD/SD, and HD/SD/HD). Housing conditions were represented in the figure parallel to the diet diagram in order to portray the temporal concurrence of these events.

### 4.3. Housing Conditions

All animals had free access to food and water and were raised under controlled room temperature (23 ± 1 °C) with a 12-h light-dark cycle. Animals were grouped in accordance with two possibilities of environmental conditions: half of them were kept in the impoverished environment of standard laboratory cages (impoverished environment (IE)) and the other half in environmental enriched cages (enriched environment (EE)). IE was composed of 32 cm × 45 cm × 16.5 cm size plastic cages for mice covered with metal grids. Each standard cage housed six mice. EE consisted of two-level wire cages each of which with 50 cm × 50 cm × 50 cm equipped with rope bridges, tunnels, rods, running wheels, and toys. The toys were made of different types of plastic and colors being displaced or replaced periodically to expose animals to different visual, motor, and somatosensory stimuli [39]. Water and food were offered on the upper and lower floors, respectively. This arrangement forced mice to move from one floor to the other to drink or eat. Each EE cage housed 12 mice. Table 3 shows how the 12 experimental groups were organized: 2 environments × 2 ages × 3 diet regimes.

### 4.4. Body Weight

To detect potential influences of diet regimes and environments on body weight, all animals were weighed at the beginning of the experiment and at the end of each time window (6M, 18M).

### 4.5. Perfusion and Histology Procedures

After completing the experimental time for each group, animals were weighed and sacrificed with an overdose of anesthetic. The perfusion technique used xylazine (0.001 mL/g) and ketamine (0.0024 mL/g), in a 1:4 ratio, as inducers of deep anesthesia. Once the anesthetic effect was obtained, a thoracotomy was performed to access the left ventricle. The animals were perfused transcardially with heparinized saline over 10 min, followed by 4% paraformaldehyde fixative (in 0.1 M phosphate buffer, pH 7.2–7.4). At the end of the procedure, brains were removed and cut on a vibratome (MicromR HM 650) at a 60µm thickness. Sections were organized as an anatomical 1:6 series of sections that were code-labeled to assure blind procedures for cell counting. One section from each of the six anatomical series and each experimental group was immunolabeled for the selective astrocyte marker glial fibrillary acidic protein (GFAP; mouse anti-GFAP monoclonal antibody MAB360, Millipore Int., Burlington, MA, USA) using free-floating immunohistochemistry.

Free-floating sections were rinsed once in 0.1 M phosphate buffer for 2 min, transferred to 0.01 M citrate buffer pH 6.0, heated to 85 °C for 1 h, and then rested in the same solution for 20 min at room temperature. Thereafter, sections were washed once in Tris-EDTA buffer for 12 min, followed by a wash of 3 × 5 min in PBST (5%). Subsequently, sections were washed for 3 × 2 min in PBS. The sections were then blocked using immunoglobulin for 1 h, following the instructions for the Mouse-on-Mouse Immunodetection Kit (M.O.M. Kit, Vector Laboratories, Newark, CA, USA). Blocking was followed by washing (3 × 2 min) in PBS. Sections were incubated in a working solution of protein concentrate for 5 min, then incubated with monoclonal mouse anti-GFAP primary antibody (MAB360, Chemicon International, Temecula, CA, USA) diluted 1:800 in protein concentrate solution (M.O.M. kit) and PBS at 4 °C for 3 days with continuous gentle agitation. Next, the sections were washed (3 × 2 min) in PBS and incubated for 12 h with biotinylated horse anti-mouse secondary antibody (M.O.M. kit), diluted 1:100 in PBS. The sections were incubated in a 0.9% hydrogen peroxide solution in methanol under constant and gentle shaking for 15 min and rinsed for 3 × 2 min in PBS. After washing (3 × 2 min) in PBS, sections were transferred to an avidin-biotin-peroxidase complex solution (ABC, Vector Laboratories, Newark, CA, USA, 1:200) for 1 h, washed (3 × 2 min) in 0.1 M PB, and rinsed once in acetate buffer 0.2 M pH 6.0 for 5 min. Finally, they were processed using the glucose oxidase-DAB-nickel method and peroxidase histochemistry (Shu et al. 1988) for a maximum of 30 min.

The reaction was interrupted when fine astrocytic branches were detected under the microscope. Sections were rinsed (3 × 2 min) in 0.1 M PB, mounted on gelatinized slides, dehydrated in alcohol and xylene, and topped by a coverslip with Enthelan^®^ (Merck©, Rahway, NJ, USA).

### 4.6. Microscopy and Optical Fractionator

**Figure 6:**
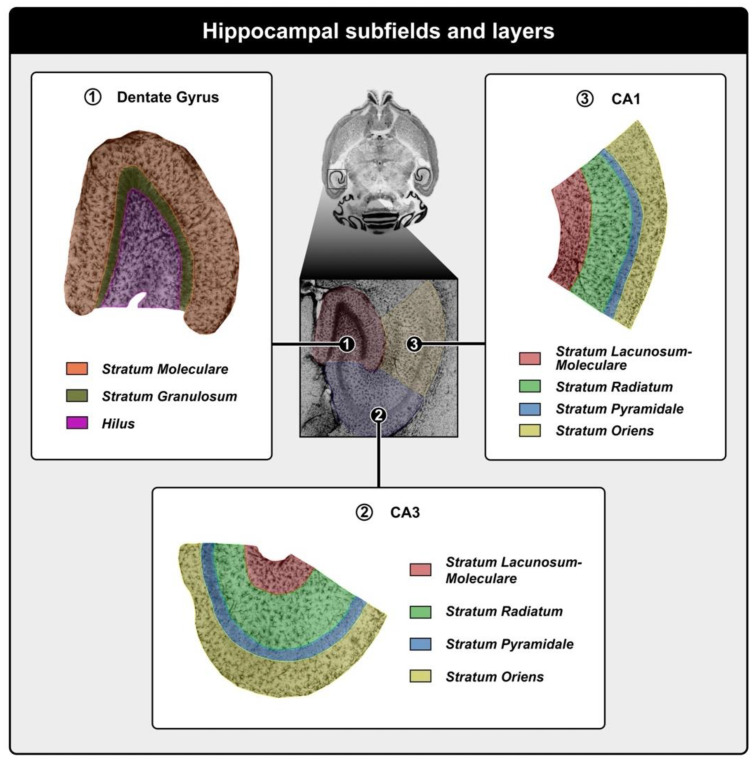
Photomicrograph of the regions of interest and analyzed layers (low-power magnitude, 4×, inset in the center). In the center, we observe a horizontal section of the *Mus musculus* mouse brain, highlighting regions of hippocampal formation immunolabeled for GFAP. They are: (**1**) Dentate gyrus: showing the stratatum moleculare and hilus, respectively, by the colors orange and pink. (**2**) CA3, containing 4 regions: Stratum lacunosum-moleculare (in red), Stratum radiatum (green), Stratum pyramidale (blue), and Stratum oriens (yellow). (**3**) CA1, containing 4 regions, homonymous to the CA3 region. The horizontal section in the center of this figure was extracted from a virtual mouse brain atlas and can be accessed via the following link: https://www.mbl.org/atlas232/atlas232_frame.html.

The total number of astrocytes in the CA1 and CA3 subfields and in the dentate gyrus (DG) was estimated using the optical fractionator technique (Figure 6). A systematic random sampling scheme was designed so that all parts of the anatomical region of interest had an equal opportunity of being sampled with optical dissectors. Cells were counted in every 6th section through the entire hippocampus using the StereoInvestigator System^®^ (Microbrightfield©, Williston, VT, USA). This system consisted of a color digital video camera that interfaced with a Nikon E600 microscope. The astrocytes were counted from randomly and systematically multiple selected frames in every serial section using a 100× oil immersion planapochromatic objective (Nikon, NA 1.4, Minato City, Japan).

In every section, the contours of CA1 and CA3 subfields and DG were first delineated using the tracing function of StereoInvestigator^®^ and the low power 4× objective of the Optiphot 2 microscope (Nikon©, Minato City, Japan). The following optical fractionator was activated, and the number and location of counting frames and counting depth for each section were determined by entering some parameters, as shown:

A computer-driven motorized stage (MAC6000, Ludl Electronic Products©, Hawthorne, NY, USA) then allowed the section to be analyzed at each counting frame location. In every counting frame location, the top and bottom of the section were set. After this, the focus was moved 2 μm deeper through the section (guard zone) to prevent counting inaccuracies due to the uneven section surface. All GFAP^+^ astrocytes that came into focus in the next 7 μm section thickness (optical dissector height) were counted if they were entirely within the counting frame or touching the upper or right side of the counting frame.

Based on the above parameters and cell counts, the StereoInvestigator^®^ program calculated the number of astrocytes per selected region using the optical fractionator formula N = 1/ssf × 1/asf × 1/tsf × ∑Q [61,62], where ssf represents the section sampling fraction; asf symbolizes the area sampling fraction; tsf, the tissue sampling fraction; and ∑Q denotes the total count of immunopositive cells sampled for each region. At least five serial sections were measured in each animal.

The calculation of the coefficient of error (CE) for the total cell count of each subject in the present study adopted the one-stage systematic sampling procedure (Schaeffer, CE) that has been used previously and validated elsewhere [63]. The CE expresses the accuracy of cell number estimates, and a value of CE < 0.05 was deemed appropriate because the variance introduced by the estimation procedure contributes only little to the observed group variance [63,64,65]. The experimental parameters for each layer (Table 4) were established in a pilot experiment and uniformly applied to all animals.

### 4.7. Photomicrographic Documentation

Digital photomicrographs were taken with the objective lens of a digital camera (Microfire; Optronics, Fremont, CA, USA) coupled to a Nikon microscope (Optiphot-2; Melville, NY, USA). Digital photomicrographs were processed with Adobe Photoshop CC 2019 software for scaling and for adjusting the levels of brightness and contrast, which were applied to the whole image (Figure 2). The selected micrographs display representative sections from each experimental group where statistically significant differences were identified. A representative section of each layer was taken from animals whose individual estimates of the number of astrocytes (N) were closest to the mean values of N for each group.

### 4.8. Statistical Analysis

All groups of animals were tested for statistical normality. Possible outliers identified based on standard deviations were eliminated from the data set. Parametric statistical analysis was used to assess the level of significance of the results from the optical fractionator by applying descriptive statistics (quantitative data), a three-way ANOVA, and the Tukey a priori test. The significance level for statistical differences was set at alpha < 0.05 (i.e., at a 95% confidence level). Statistical analyses were performed using BioEstat^®^ 5.0, Excel for Windows^®^, and GraphPad Prism 9 for Windows^®^.

## 5. Conclusions

Overall, it is possible to identify the expansion and importance of research involving neuro-responsiveness under the influence of masticatory function. In particular, we focused on the laminar sensitivity of three hippocampal subfields. The subfield CA1, as a congruent region of hippocampal outputs from the trisynaptic circuit and the temporo-ammonic pathway, actually showed sensitivity with the Stratum radiatum responding to the variables age and diet regimes, where age suggests a loss of astrocytic population and the enriched environment guiding the population in a sense close to that of the control animals in practically all other age groups through masticatory activity and environmental stimulation. The possibility of the astrocytic population inciting other molecular, morphological, or cellular responses in an integrated way offers a vast amount of need for studies, along with the possibilities of coping with the dysfunction of masticatory activity and its restoration.

## Figures and Tables

**Table 1:** The mean and standard error of the body weight (g) of animals for each experimental group based on diet regimen (HD, HD/SD, and HD/SD/HD), age (6 or 18 months), and both environments (impoverished or enriched).

	Body Weight (g): Mean ± Standard Error			
	Impoverished Environment (IE)		Enriched Environment (EE)	
	6 Months	18 Months	6 Months	18 Months
HD	75.96 ± 3.69	75.32 ± 1.45	50.94 ± 1.25	65.21 ± 4.41
HD/SD	55.04 ± 2.05	53.44 ± 3.61	44.32 ± 3.31	51.21 ± 2.67
HD/SD/HD	71.56 ± 2.93	57.65 ± 4.10	51.70 ± 5.27	54.85 ± 2.09

**Table 2:** Significance values for comparisons between the body weight of animals in each experimental group based on diet regime (HD, HD/SD, and HD/SD/HD), age (6 or 18 months), and both environments (impoverished or enriched).

	Statistical Significance Values			
	Impoverished Environment (IE)		Enriched Environment (EE)	
	6 Months	18 Months	6 Months	18 Months
ANOVA-one way	F_(2,12)_ = 13.83 *p* = 0.01	F_(2,12)_ = 12.63 *p* = 0.0014	F_(2,12)_ = 1.23 *p* = 0.328	F_(2,12)_ = 5.10 *p* = 0.025
HD vs. HD/SD	t_(8)_ = 4.99 *p* < 0.001	t_(8)_ = 4.74 *p* < 0.001	-	t_(8)_ = 3.08 *p* = 0.0095
HD vs. HD/SD/HD	t_(8)_ = 1.05 *p* > 0.05	t_(8)_ = 3.82 *p* = 0.0024	-	t_(8)_ = 2.28 *p* < 0.0418
HD/SD vs. HD/SD/HD	t_(8)_ = 3.94 *p* = 0.002	t_(8)_ = 0.91 *p* > 0.05	-	t_(8)_ = 0.80 *p* > 0.05

**Table 3:** Experimental groups and the distribution of the number of animals used in each group. Distribution of the number of animals per diet regimen (HD, HD/SD, and HD/SD/HD), in impoverished (IE) and enriched (EE) environments, at the ages of 6- and 18-month-old.

Ages	Number of Animals					
	Impoverished Environment (IE)			Enriched Environment (EE)		
	HD	HD/SD	HD/SD/HD	HD	HD/SD	HD/SD/HD
6-month	5	5	5	5	5	5
18-month	5	5	5	5	5	5

**Table 4:** Stereological parameters of the quantitative estimate of CA1, CA3, and DG of mice submitted to mastication dysfunction, an enriched environment, and aging.

Region	Layer	Counting Frame Size (μm × μm)	Grid Size (μm × μm)	Guard Zone (μm)	Optical Dissector Height (μm)
CA1	LM	80 × 80	80 × 80	2	7
	RAD	80 × 80	80 × 80		
	OR	80 × 80	80 × 80		
CA3	LM	40 × 40	50 × 50		
	RAD	80 × 80	80 × 80		
	OR	80 × 80	80 × 80		
Dentate Gyrus	MOL	80 × 80	80 × 80		
	HILUS	80 × 80	80 × 80		

LM—Stratum Lacunosum-moleculare; RAD—Stratum Radiatum; OR—Stratum Oriens; MOL—Molecular; HILUS.

## Data Availability

The original contributions generated for this study are included in the article/Supplementary Materials. Further inquiries can be directed to the corresponding author.

## Supplementary Materials

The following supporting information can be downloaded at: https://www.mdpi.com/article/10.3390/ijms24076529/s1.
