# Supplementary material for: The Masticatory Activity Interference in Quantitative Estimation of CA1, CA3 and Dentate Gyrus Hippocampal Astrocytes of Aged Murine Models and under Environmental Stimulation
Source: Int J Mol Sci. 2023 Mar 31;24(7):6529. doi: 10.3390/ijms24076529 (PMC10095286; doi:10.3390/ijms24076529)
Supplement: Supplementary file 1 [file ijms-24-06529-s001.zip › ijms-2223210-supplementary.pdf]

Table S1. Estimated Unilateral Numbers of Astrocytes (N) With the Coefficient of Error (CE) for the Stratum Lacunosum-Moleculare of CA1 of 6-, and 18-Month-Old Female Albino Swiss Mice Fed a Hard Diet (HD), Hard/Soft Diet (HD/SD) and Hard/Soft/Hard Diet (HDSDHD).

STRATUM LACUNOSUM-MOLECULARE – CA1			
**HD / 6M / Impoverished Environment**			
**Subjects**	**N**	**Thickness (µm)**	**CE (Scheaffer)**
HD 6M IE Animal 1	10847.06	24.77 ± 0.76	0.05
HD 6M IE Animal 2	12047.31	23.4 ± 0.68	0.06
HD 6M IE Animal 3	24290.66	21.74 ± 0.65	0.04
HD 6M IE Animal 4	11168.4	25.27 ± 0.42	0.05
HD 6M IE Animal 5	15503.83	23.33 ± 0.68	0.05
Mean	14771.45	23.71 ± 0.64	0.05
Standard Error	2519.48	0.62 ± 0.06	
CV^2	0.145		
CE^2	0.002		
CE^2 / CV^2	0.017		
CVB^2	0.143		
CVB^2 (% of CV^2)	98.35%		
**HDSD / 6M / Impoverished Environment**			
**Subjects**	**N**	**Thickness (µm)**	**CE (Scheaffer)**
HDSD 6M IE Animal 1	8634.51	26.00 ± 0.48	0.05
HDSD 6M IE Animal 2	11173.63	23.61 ± 1.14	0.05
HDSD 6M IE Animal 3	7505.31	21.53 ± 0.77	0.05
HDSD 6M IE Animal 4	10758.6	22.39 ± 0.79	0.05
HDSD 6M IE Animal 5	13272.69	24.01 ± 0.37	0.05
Mean	10268.95	23.51 ± 0.71	0.05
Standard Error	1009.70	0.76 ± 0.13	
CV^2	0.048		
CE^2	0.002		
CE^2 / CV^2	0.054		
CVB^2	0.046		
CVB^2 (% of CV^2)	94.51%		

HDSDHD / 6M / Impoverished Environment			
**Subjects**	**N**	**Thickness (µm)**	**CE (Scheaffer)**
HDSDHD 6M IE Animal 1	17495.57	23.88 ± 0.82	0.05
HDSDHD 6M IE Animal 2	9147.94	21.67 ± 0.23	0.06
HDSDHD 6M IE Animal 3	14870.31	21.21 ± 0.49	0.05
HDSDHD 6M IE Animal 4	13456.29	22.67 ± 0.65	0.05
HDSDHD 6M IE Animal 5	9521.06	20.40 ± 0.79	0.05
Mean	12898.23	21.97 ± 0.60	0.05
Standard Error	1593.83	0.60 ± 0.11	
CV^2	0.076		
CE^2	0.002		
CE^2 / CV^2	0.033		
CVB^2	0.074		
CVB^2 (% of CV^2)	96.74%		
**HD / 18M / Impoverished Environment**			
**Subjects**	**N**	**Thickness (µm)**	**CE (Scheaffer)**
HD 18M IE Animal 1	7696.28	25.41 ± 0.39	0.06
HD 18M IE Animal 2	9894.43	26.34 ± 0.35	0.06
HD 18M IE Animal 3	11195.23	25.80 ± 0.27	0.06
HD 18M IE Animal 4	6424.63	23.2 ± 0.56	0.06
HD 18M IE Animal 5	6650.14	23.2 ± 0.2	0.07
Mean	8372.14	24.79 ± 0.35	0.06
Standard Error	935.47	0.67 ± 0.06	
CV^2	0.062		
CE^2	0.003		
CE^2 / CV^2	0.056		
CVB^2	0.059		
CVB^2 (% of CV^2)	94.38%		
**HDSD / 18M / Impoverished Environment**			
**Subjects**	**N**	**Thickness (µm)**	**CE (Scheaffer)**
HDSD 18M IE Animal 1	10726.71	24.25 ± 0.46	0.05
HDSD 18M IE Animal 2	8131.89	23.91 ± 0.68	0.06
HDSD 18M IE Animal 3	8522.65	22.58 ± 0.51	0.06
HDSD 18M IE Animal 4	5979.69	26.19 ± 0.91	0.06

HDSD 18M IE Animal 5	16286.57	26.04 ± 0.27	0.05
Mean	9929.50	24.60 ± 0.56	0.06
Standard Error	1758.69	0.68 ± 0.11	
CV^2	0.157		
CE^2	0.003		
CE^2 / CV^2	0.021		
CVB^2	0.154		
CVB^2 (% of CV^2)	97.93%		
**HDSDHD / 18M / Impoverished Environment**			
**Subjects**	**N**	**Thickness (µm)**	**CE (Scheaffer)**
HDSDHD 18M IE Animal 1	18148.54	26.56 ± 1.10	0.04
HDSDHD 18M IE Animal 2	13046.57	24.53 ± 0.92	0.05
HDSDHD 18M IE Animal 3	8379.60	21.99 ± 0.59	0.05
HDSDHD 18M IE Animal 4	16134.17	24.01 ± 0.61	0.05
HDSDHD 18M IE Animal 5	12367.11	23.60 ± 1.18	0.05
Mean	13615.20	24.14 ± 0.88	0.05
Standard Error	1676.14	0.74 ± 0.12	
CV^2	0.076		
CE^2	0.003		
CE^2 / CV^2	0.034		
CVB^2	0.073		
CVB^2 (% of CV^2)	96.60%		
**HD / 6M / Enriched Environment**			
**Subjects**	**N**	**Thickness (µm)**	**CE (Scheaffer)**
HD 6M EE Animal 1	16077.43	25.54 ± 0.42	0.04
HD 6M EE Animal 2	10361.74	21.72 ± 0.42	0.06
HD 6M EE Animal 3	13856.49	26.45 ± 0.45	0.04
HD 6M EE Animal 4	15499.29	25.71 ± 1.09	0.04
HD 6M EE Animal 5	17044.8	23.92 ± 0.67	0.04
Mean	14567.95	24.67 ± 0.61	0.05
Standard Error	1172.16	0.84 ± 0.13	
CV^2	0.032		
CE^2	0.002		
CE^2 / CV^2	0.065		

CVB^2	0.065		
CVB^2 (% of CV^2)	93.55%		
**HDSD / 6M / Enriched Environment**			
**Subjects**	**N**	**Thickness (μm)**	**CE (Scheaffer)**
HDSD 6M EE Animal 1	14118	25.62 ± 0.95	0.05
HDSD 6M EE Animal 2	11958.42	22.10 ± 0.72	0.05
HDSD 6M EE Animal 3	16099.03	24.03 ± 0.89	0.05
HDSD 6M EE Animal 4	21209.57	23.95 ± 0.71	0.04
HDSD 6M EE Animal 5	18310.63	27.75 ± 0.30	0.05
Mean	16339.13	24.69 ± 0.71	0.05
Standard Error	1609.19	0.95 ± 0.11	
CV^2	0.048		
CE^2	0.002		
CE^2 / CV^2	0.043		
CVB^2	0.046		
CVB^2 (% of CV^2)	95.67%		
**HDSDHD / 6M / Enriched Environment**			
**Subjects**	**N**	**Thickness (μm)**	**CE (Scheaffer)**
HDSDHD 6M EE Animal 1	15701.83	21.08 ± 0.73	0.05
HDSDHD 6M EE Animal 2	14057.57	22.16 ± 0.5	0.04
HDSDHD 6M EE Animal 3	12307.11	24.38 ± 0.69	0.05
HDSDHD 6M EE Animal 4	13913.14	24.71 ± 0.5	0.05
HDSDHD 6M EE Animal 5	15292.46	26.56 ± 0.43	0.05
Mean	14254.42	23.78 ± 0.58	
Standard Error	596.65	0.97 ± 0.06	
CV^2	0.009		
CE^2	0.002		
CE^2 / CV^2	0.262		
CVB^2	0.006		
CVB^2 (% of CV^2)	73.808%		
**HD / 18M / Enriched Environment**			
**Subjects**	**N**	**Thickness (μm)**	**CE (Scheaffer)**
HD 18M EE Animal 1	19155.86	25.78 ± 0.73	0.05
HD 18M EE Animal 2	14752.97	26.29 ± 0.36	0.05

HD 18M EE Animal 3	14111.49	27.78 ± 0.58	0.05
HD 18M EE Animal 4	16170.17	25.26 ± 0.52	0.04
HD 18M EE Animal 5	10140.51	26.56 ± 1.73	0.06
Mean	14866.20	26.33 ± 0.79	
Standard Error	1466.56	0.42 ± 0.24	
CV^2	0.049		
CE^2	0.002		
CE^2 / CV^2	0.049		
CVB^2	0.046		
CVB^2 (% of CV^2)	95.126%		
**HDSD / 18M / Enriched Environment**			
**Subjects**	**N**	**Thickness (μm)**	**CE (Scheaffer)**
HDSD 18M EE Animal 1	18938.43	24.53 ± 0.72	0.04
HDSD 18M EE Animal 2	17252.57	25.82 ± 0.62	0.04
HDSD 18M EE Animal 3	11189.49	25.29 ± 0.69	0.05
HDSD 18M EE Animal 4	16946.74	25.40 ± 0.32	0.05
HDSD 18M EE Animal 5	18383.83	26.13 ± 0.70	0.04
Mean	16542.21	25.43 ± 0.61	
Standard Error	1386.60	0.27 ± 0.07	
CV^2	0.035		
CE^2	0.002		
CE^2 / CV^2	0.056		
CVB^2	0.033		
CVB^2 (% of CV^2)	94.363%		
**HDSDHD / 18M / Enriched Environment**			
**Subjects**	**N**	**Thickness (μm)**	**CE (Scheaffer)**
HDSDHD 18M EE Animal 1	19202.06	26.41 ± 0.89	0.04
HDSDHD 18M EE Animal 2	14545.8	26.62 ± 0.93	0.05
HDSDHD 18M EE Animal 3	19602.77	24.4 ± 0.51	0.04
HDSDHD 18M EE Animal 4	12609.86	23.93 ± 0.74	0.04
HDSDHD 18M EE Animal 5	19395.51	25.37 ± 0.43	0.04
Mean	17071.20	25.35 ± 0.7	
Standard Error	1460.02	0.53 ± 0.1	
CV^2	0.037		

CE^2	0.002		
CE^2 / CV^2	0.050		
CVB^2	0.035		
CVB^2 (% of CV^2)	95.04%		

$CVB^2 = CV^2 - CE^2$ (CV, coefficient of variation; CVB, biological coefficient of variation; CE, coefficient of error). N = number of astrocytes; Mean = mean numbers in each group; 6M and 18M indicate 6 months old and 18 months old, respectively.

Table S2. Estimated Unilateral Numbers of Astrocytes (N) With the Coefficient of Error (CE) for the Stratum Radiatum of CA1 of 6-, and 18-Month-Old Female Albino Swiss Mice Fed a Hard Diet (HD), Hard/Soft Diet (HD/SD) and Hard/Soft/Hard Diet (HDSDHD).

**STRATUM RADIATUM – CA1**			
**HD / 6M / Impoverished Environment**			
**Subjects**	**N**	**Thickness (µm)**	**CE (Scheaffer)**
HD 6M IE Animal 1	13379.6	23.52 ± 1.39	0.04
HD 6M IE Animal 2	14269.4	24.66 ± 0.51	0.04
HD 6M IE Animal 3	21801.6	22.04 ± 0.99	0.04
HD 6M IE Animal 4	17233.97	24.89 ± 0.45	0.04
HD 6M IE Animal 5	15100.6	22.99 ± 0.57	0.04
Mean	16357.03	23.62 ± 0.78	0.04
Standard Error	1503.57	0.53 ± 0.18	
CV^2	0.042		
CE^2	0.001		
CE^2 / CV^2	0.033		
CVB^2	0.040		
CVB^2 (% of CV^2)	96.65%		

HDSD / 6M / Impoverished Environment			
Subjects	N	Thickness (µm)	CE (Scheaffer)
HDSD 6M IE Animal 1	8411.31	22.31 ± 0.99	0.05
HDSD 6M IE Animal 2	12215.57	23.43 ± 0.94	0.04
HDSD 6M IE Animal 3	9707.06	21.635 ± 0.70	0.04
HDSD 6M IE Animal 4	11952	21.03 ± 0.60	0.03
HDSD 6M IE Animal 5	13537.8	24.17 ± 0.33	0.04
Mean	11164.75	22.52 ± 0.71	0.04
Standard Error	923.62	0.57 ± 0.12	
CV^2	0.034		
CE^2	0.002		
CE^2 / CV^2	0.050		
CVB^2	0.032		
CVB^2 (% of CV^2)	94.92%		
HDSDHD / 6M / Impoverished Environment			
Subjects	N	Thickness (µm)	CE (Scheaffer)
HDSDHD 6M IE Animal 1	15494.57	23.27 ± 1.08	0.04
HDSDHD 6M IE Animal 2	10713.94	21.49 ± 0.4	0.04
HDSDHD 6M IE Animal 3	14380.03	20.77 ± 0.62	0.03
HDSDHD 6M IE Animal 4	14769.43	22.70 ± 0.45	0.04
HDSDHD 6M IE Animal 5	11700.77	19.66 ± 0.75	0.04
Mean	13411.75	21.58 ± 0.66	0.04
Standard Error	930.72	0.65 ± 0.12	
CV^2	0.024		
CE^2	0.001		
CE^2 / CV^2	0.058		
CVB^2	0.023		
CVB^2 (% of CV^2)	94.22%		
HD / 18M / Impoverished Environment			
Subjects	N	Thickness (µm)	CE (Scheaffer)
HD 18M IE Animal 1	10310.57	24.52 ± 0.42	0.04
HD 18M IE Animal 2	8713.37	23.84 ± 0.32	0.05
HD 18M IE Animal 3	8809.37	26.14 ± 0.46	0.05
HD 18M IE Animal 4	8966.06	23.51 ± 0.60	0.05

HD 18M IE Animal 5	8701.46	23 ± 0.45	0.05
Mean	9100.17	24.20 ± 0.45	0.05
Standard Error	306.28	0.54 ± 0.04	
CV^2	0.006		
CE^2	0.002		
CE^2 / CV^2	0.390		
CVB^2	0.003		
CVB^2 (% of CV^2)	61%		
**HDSD / 18M / Impoverished Environment**			
**Subjects**	**N**	**Thickness (µm)**	**CE (Scheaffer)**
HDSD 18M IE Animal 1	9728.314	21.68 ± 0.38	0.04
HDSD 18M IE Animal 2	10257.77	23.54 ± 0.54	0.04
HDSD 18M IE Animal 3	9624.171	22.37 ± 0.37	0.04
HDSD 18M IE Animal 4	14611.54	25.74 ± 0.37	0.05
HDSD 18M IE Animal 5	18124.54	25.74 ± 0.41	0.05
Mean	12469.27	23.81 ± 0.41	0.04
Standard Error	1689.22	0.84 ± 0.03	
CV^2	0.092		
CE^2	0.002		
CE^2 / CV^2	0.022		
CVB^2	0.090		
CVB^2 (% of CV^2)	97.82%		
**HDSDHD / 18M / Impoverished Environment**			
**Subjects**	**N**	**Thickness (µm)**	**CE (Scheaffer)**
HDSDHD 18M IE Animal 1	17217.94	26.24 ± 1.14	0.05
HDSDHD 18M IE Animal 2	14079.60	23.58 ± 0.96	0.04
HDSDHD 18M IE Animal 3	13288.20	21.68 ± 0.63	0.03
HDSDHD 18M IE Animal 4	17629.63	24.01 ± 1.04	0.05
HDSDHD 18M IE Animal 5	15056.23	23.12 ± 1.05	0.04
Mean	15454.32	23.73 ± 0.96	0.04
Standard Error	853.90	0.74 ± 0.09	
CV^2	0.015		
CE^2	0.002		

CE^2 / CV^2	0.113		
CVB^2	0.014		
CVB^2 (% of CV^2)	88.69%		
**HD / 6M / Enriched Environment**			
**Subjects**	**N**	**Thickness (µm)**	**CE (Scheaffer)**
HD 6M IE Animal 1	14028.09	24.26 ± 0.60	0.04
HD 6M IE Animal 2	11639.23	21.45 ± 0.27	0.04
HD 6M IE Animal 3	12593.31	26.20 ± 0.57	0.04
HD 6M IE Animal 4	14745.17	24.25 ± 1.05	0.04
HD 6M IE Animal 5	14787.17	23.96 ± 0.41	0.03
Mean	13558.59	24.02 ± 0.58	0.04
Standard Error	622.64	0.76 ± 0.13	
CV^2	0.010		
CE^2	0.001		
CE^2 / CV^2	0.132		
CVB^2	0.009		
CVB^2 (% of CV^2)	86.81%		
**HDSD / 6M / Enriched Environment**			
**Subjects**	**N**	**Thickness (µm)**	**CE (Scheaffer)**
HDSD 6M IE Animal 1	12729.43	25.51 ± 0.97	0.04
HDSD 6M IE Animal 2	13585.63	22.26 ± 0.59	0.04
HDSD 6M IE Animal 3	13139.66	23.74 ± 1.00	0.03
HDSD 6M IE Animal 4	18052.8	24.25 ± 0.87	0.03
HDSD 6M IE Animal 5	16848.6	27.20 ± 0.53	0.04
Mean	14871.22	24.59 ± 0.79	0.04
Standard Error	1078.68	0.83 ± 0.1	
CV^2	0.030		
CE^2	0.001		
CE^2 / CV^2	0.052		
CVB^2	0.025		
CVB^2 (% of CV^2)	94.8%		
**HDSDHD / 6M / Enriched Environment**			
**Subjects**	**N**	**Thickness (µm)**	**CE (Scheaffer)**
HDSDHD 6M IE Animal 1	15753	21 ± 0.9	0.04

HDSDHD 6M IE Animal 2	10886.83	21.08 ± 0.24	0.04
HDSDHD 6M IE Animal 3	11038.9	24.24 ± 0.56	0.04
HDSDHD 6M IE Animal 4	11992.8	25.31 ± 0.22	0.04
HDSDHD 6M IE Animal 5	12601.2	25.37 ± 0.5	0.04
Mean	12454.5	23.4 ± 0.49	0.04
Standard Error	882.5	0.99 ± 0.12	
CV^2	0.025		
CE^2	0.002		
CE^2 / CV^2	0.064		
CVB^2	0.023		
CVB^2 (% of CV^2)	93.6%		
**HD / 18M / Enriched Environment**			
**Subjects**	**N**	**Thickness (μm)**	**CE (Scheaffer)**
HD 18M IE Animal 1	12827.5	25.77 ± 0.91	0.05
HD 18M IE Animal 2	11742.9	25.24 ± 0.66	0.04
HD 18M IE Animal 3	11182.8	26.92 ± 0.23	0.05
HD 18M IE Animal 4	13611.3	24.58 ± 0.58	0.04
HD 18M IE Animal 5	11620.4	26.37 ± 1.62	0.04
Mean	12196.97	25.77 ± 0.8	0.04
Standard Error	445.3	0.41 ± 0.23	
CV^2	0.007		
CE^2	0.002		
CE^2 / CV^2	0.300		
CVB^2	0.005		
CVB^2 (% of CV^2)	71.08%		
**HDSD / 18M / Enriched Environment**			
**Subjects**	**N**	**Thickness (μm)**	**CE (Scheaffer)**
HDSD 18M IE Animal 1	14610.6	24.3 ± 0.39	0.03
HDSD 18M IE Animal 2	20168.91	26.11 ± 0.44	0.03
HDSD 18M IE Animal 3	9718.11	25.3 ± 0.58	0.05
HDSD 18M IE Animal 4	16181.83	25.27 ± 0.48	0.03
HDSD 18M IE Animal 5	15019.63	26 ± 0.67	0.04
Mean	15139.82	25.39 ± 0.51	0.04
Standard Error	1674.32	0.33 ± 0.05	

CV^2	0.153		
CE^2	0.004		
CE^2 / CV^2	0.024		
CVB^2	0.149		
CVB^2 (% of CV^2)	97.6%		
**HDSDHD / 18M / Enriched Environment**			
**Subjects**	**N**	**Thickness (µm)**	**CE (Scheaffer)**
HDSDHD 18M IE Animal 1	13603.63	25.96 ± 0.71	0.04
HDSDHD 18M IE Animal 2	11730.09	26.01 ± 0.57	0.04
HDSDHD 18M IE Animal 3	15370.2	24.15 ± 0.57	0.03
HDSDHD 18M IE Animal 4	11822.4	23.79 ± 0.64	0.04
HDSDHD 18M IE Animal 5	17941.54	25.36 ± 0.63	0.03
Mean	14093.57	25.05 ± 0.62	0.04
Standard Error	1170.92	0.46 ± 0.03	
CV^2	0.035		
CE^2	0.001		
CE^2 / CV^2	0.039		
CVB^2	0.033		
CVB^2 (% of CV^2)	96.09%		

$CVB^2 = CV^2 - CE^2$ (CV, coefficient of variation; CVB, biological coefficient of variation; CE, coefficient of error). N = number of astrocytes; Mean = mean numbers in each group; 6M and 18M indicate 6 months old and 18 months old, respectively.

Table S3. Estimated Unilateral Numbers of Astrocytes (N) With the Coefficient of Error (CE) for the Stratum Oriens of CA1 of 6-, and 18-Month-Old Female Albino Swiss Mice Fed a Hard Diet (HD), Hard/Soft Diet (HD/SD) and Hard/Soft/Hard Diet (HDSDHD).

STRATUM ORIENS – CA1			
HD / 6M / Impoverished Environment			
Subjects	N	Thickness (µm)	CE (Scheaffer)
HD 6M IE Animal 1	7139.47	23.01 ± 1.96	0.05
HD 6M IE Animal 2	12993.6	24.62 ± 0.40	0.04
HD 6M IE Animal 3	17970.6	22.21 ± 0.50	0.03
HD 6M IE Animal 4	12052.54	24.43 ± 0.46	0.04
HD 6M IE Animal 5	10467.6	22.69 ± 0.55	0.04
Mean	12124.77	23.39 ± 0.78	0.05
Standard Error	1768.23	0.48 ± 0.30	
CV^2	0.106		
CE^2	0.002		
CE^2 / CV^2	0.020684		
CVB^2	0.104		
CVB^2 (% of CV^2)	97.93%		
HDSD / 6M / Impoverished Environment			
Subjects	N	Thickness (µm)	CE (Scheaffer)
HDSD 6M IE Animal 1	8268.43	22.53 ± 1.17	0.05
HDSD 6M IE Animal 2	11327.14	23.82 ± 1.03	0.05
HDSD 6M IE Animal 3	6915.43	21.79 ± 0.71	0.05
HDSD 6M IE Animal 4	9443.31	22.60 ± 0.29	0.04
HDSD 6M IE Animal 5	11758.46	24.96 ± 0.26	0.04
Mean	9542.55	23.14 ± 0.70	0.05
Standard Error	911.87	0.56 ± 0.19	
CV^2	0.046		
CE^2	0.002		
CE^2 / CV^2	0.050		
CVB^2	0.043		
CVB^2 (% of CV^2)	94.97%		
HDSDHD / 6M / Impoverished Environment			
Subjects	N	Thickness (µm)	CE (Scheaffer)
HDSDHD 6M IE Animal 1	15625.71	24.55 ± 1.07	0.04
HDSDHD 6M IE Animal 2	7799.06	21.78 ± 0.44	0.05
HDSDHD 6M IE Animal 3	11307.34	21.54 ± 0.72	0.04

HDSDHD 6M IE Animal 4	10604.91	23.41 ± 0.49	0.05
HDSDHD 6M IE Animal 5	8830.11	20.26 ± 0.94	0.05
Mean	10833.43	22.31 ± 0.73	0.05
Standard Error	1350.25	0.75 ± 0.12	
CV^2	0.077		
CE^2	0.002		
CE^2 / CV^2	0.029		
CVB^2	0.075		
CVB^2 (% of CV^2)	97.14%		
**HD / 18M / Impoverished Environment**			
**Subjects**	**N**	**Thickness (µm)**	**CE (Scheaffer)**
HD 18M IE Animal 1	7948.87	24.86 ± 0.53	0.05
HD 18M IE Animal 2	9246.07	25.58 ± 0.50	0.06
HD 18M IE Animal 3	8324.66	26.54 ± 0.39	0.06
HD 18M IE Animal 4	5981.14	24.36 ± 0.11	0.06
HD 18M IE Animal 5	7076.57	23.8 ± 0.37	0.06
Mean	7715.47	25.03 ± 0.38	0.06
Standard Error	556.08	0.48 ± 0.07	0.05
CV^2	0.026		
CE^2	0.003		
CE^2 / CV^2	0.130		
CVB^2	0.023		
CVB^2 (% of CV^2)	87%		
**HDSD / 18M / Impoverished Environment**			
**Subjects**	**N**	**Thickness (µm)**	**CE (Scheaffer)**
HDSD 18M IE Animal 1	6024.51	21.98 ± 0.28	0.06
HDSD 18M IE Animal 2	8267.31	25.11 ± 0.36	0.05
HDSD 18M IE Animal 3	7449.51	22.89 ± 0.37	0.05
HDSD 18M IE Animal 4	18419.83	26.31 ± **0.51**	0.05
HDSD 18M IE Animal 5	20229.94	26.13 ± **0.29**	0.04
Mean	12078.22	24.49 ± 0.36	0.05
Standard Error	2993.84	0.87 ± 0.04	
CV^2	0.307		
CE^2	0.003		

CE^2 / CV^2	0.009		
CVB^2	0.304		
CVB^2 (% of CV^2)	99.11%		
**HDSDHD / 18M / Impoverished Environment**			
**Subjects**	**N**	**Thickness (µm)**	**CE (Scheaffer)**
HDSDHD 18M IE Animal 1	16090.29	26.86 ± 0.95	0.05
HDSDHD 18M IE Animal 2	12443.14	25.05 ± 1.01	0.05
HDSDHD 18M IE Animal 3	8401.37	22.03 ± 0.72	0.05
HDSDHD 18M IE Animal 4	17359.29	176.86 ± 151.76	0.05
HDSDHD 18M IE Animal 5	10174.54	23.93 ± 0.95	0.05
Mean	12893.73	54.95 ± 31.08	0.05
Standard Error	1702.02	30.49 ± 30.17	0.05
CV^2	0.087		
CE^2	0.002		
CE^2 / CV^2	0.027		
CVB^2	0.085		
CVB^2 (% of CV^2)	97.32%		
**HD / 6M / Enriched Environment**			
**Subjects**	**N**	**Thickness (µm)**	**CE (Scheaffer)**
HD 6M EE Animal 1	13084.29	23.62 ± 0.32	0.04
HD 6M EE Animal 2	7827.943	22.41 ± 0.48	0.05
HD 6M EE Animal 3	13229.31	26.65 ± 0.36	0.04
HD 6M EE Animal 4	11662.03	25.80 ± 0.92	0.05
HD 6M EE Animal 5	15107.31	25.31 ± 0.64	0.04
Mean	12182.18	24.76 ± 0.54	0.04
Standard Error	1218.54	0.77 ± 0.11	
CV^2	0.050		
CE^2	0.002		
CE^2 / CV^2	0.039		
CVB^2	0.048		
CVB^2 (% of CV^2)	96.07%		
**HDSD / 6M / Enriched Environment**			
**Subjects**	**N**	**Thickness (µm)**	**CE (Scheaffer)**

HDSD 6M EE Animal 1	9346.11	25.61 ± 0.86	0.06
HDSD 6M EE Animal 2	9700.03	22.60 ± 0.55	0.05
HDSD 6M EE Animal 3	10650.69	23.61 ± 0.93	0.05
HDSD 6M EE Animal 4	15628.97	24.86 ± 0.75	0.04
HDSD 6M EE Animal 5	13275.86	27.47 ± 0.43	0.05
Mean	11720.33	24.83 ± 0.70	0.05
Standard Error	1194.99	0.84 ± 0.09	
CV^2	0.052		
CE^2	0.003		
CE^2 / CV^2	0.051		
CVB^2	0.049		
CVB^2 (% of CV^2)	94.94%		
**HDSDHD / 6M / Enriched Environment**			
**Subjects**	**N**	**Thickness (µm)**	**CE (Scheaffer)**
HDSDHD 6M EE Animal 1	9493.02	21.18 ± 0.77	0.05
HDSDHD 6M EE Animal 2	7589.31	21.24 ± 0.52	0.05
HDSDHD 6M EE Animal 3	8884.97	24.54 ± 0.28	0.05
HDSDHD 6M EE Animal 4	10250.49	25.53 ± 0.41	0.06
HDSDHD 6M EE Animal 5	11534.14	25.00 ± 0.32	0.05
Mean	9550.39	23.50 ± 0.46	0.05
Standard Error	660.20	0.95 ± 0.09	
CV^2	0.024		
CE^2	0.003		
CE^2 / CV^2	0.111		
CVB^2	0.021		
CVB^2 (% of CV^2)	88.94%		
**HD / 18M / Enriched Environment**			
**Subjects**	**N**	**Thickness (µm)**	**CE (Scheaffer)**
HD 18M EE Animal 1	11814.69	26.50 ± 1.06	0.06
HD 18M EE Animal 2	10945.37	25.63 ± 0.72	0.05
HD 18M EE Animal 3	8336.14	25.46 ± 0.22	0.05
HD 18M EE Animal 4	9057.17	24.62 ± 0.58	0.05
HD 18M EE Animal 5	8474.57	26.71 ± 1.31	0.05
Mean	9725.59	25.79 ± 0.78	0.05

Standard Error	699.81	0.38 ± 0.19	
CV^2	0.026		
CE^2	0.003		
CE^2 / CV^2	0.11		
CVB^2	0.023		
CVB^2 (% of CV^2)	89.03%		
**HDSD / 18M / Enriched Environment**			
**Subjects**	**N**	**Thickness (μm)**	**CE (Scheaffer)**
HDSD 18M EE Animal 1	12923.31	24.62 ± 0.56	0.04
HDSD 18M EE Animal 2	18113.40	26.84 ± 0.19	0.04
HDSD 18M EE Animal 3	8500.29	25.77 ± 0.17	0.05
HDSD 18M EE Animal 4	12517.20	25.24 ± 0.65	0.04
HDSD 18M EE Animal 5	12929.74	26.76 ± 0.28	0.05
Mean	12996.79	25.85 ± 0.37	0.04
Standard Error	1527.06	0.43 ± 0.1	
CV^2	0.069		
CE^2	0.002		
CE^2 / CV^2	0.03		
CVB^2	0.067		
CVB^2 (% of CV^2)	97.12%		
**HDSDHD / 18M / Enriched Environment**			
**Subjects**	**N**	**Thickness (μm)**	**CE (Scheaffer)**
HDSDHD 18M EE Animal 1	13448.23	25.91 ± 0.70	0.04
HDSDHD 18M EE Animal 2	8800.63	26.66 ± 0.66	0.05
HDSDHD 18M EE Animal 3	12913.71	23.75 ± 0.67	0.04
HDSDHD 18M EE Animal 4	8741.74	24.21 ± 1.06	0.05
HDSDHD 18M EE Animal 5	13401.60	24.95 ± 0.55	0.04
Mean	11461.18	25.09 ± 0.73	0.04
Standard Error	1102.21	0.54 ± 0.09	
CV^2	0.046		
CE^2	0.002		
CE^2 / CV^2	0.043		
CVB^2	0.044		
CVB^2 (% of CV^2)	95.66%		

$CVB^2 = CV^2 - CE^2$ (CV, coefficient of variation; CVB, biological coefficient of variation; CE, coefficient of error). N = number of astrocytes; Mean = mean numbers in each group; 6M and 18M indicate 6 months old and 18 months old, respectively.

Table S4. Estimated Unilateral Numbers of Astrocytes (N) With the Coefficient of Error (CE) for the Stratum Lacunosum-Moleculare of CA3 of 6-, and 18-Month-Old Female Albino Swiss Mice Fed a Hard Diet (HD), Hard/Soft Diet (HD/SD) and Hard/Soft/Hard Diet (HDSDHD).

STRATUM LACUNOSUM-MOLECULARE – CA3			
**HD / 6M / Impoverished Environment**			
**Subjects**	**N**	**Thickness (μm)**	**CE (Scheaffer)**
HD 6M IE Animal 1	5033.44	32.51 ± 1.38	0.10
HD 6M IE Animal 2	6175.80	21.71 ± 0.26	0.06
HD 6M IE Animal 3	6952.50	25.18 ± 0.41	0.06
HD 6M IE Animal 4	7842.05	34.72 ± 1.07	0.06
HD 6M IE Animal 5	5918.57	25.46 ± 0.61	0.07
Mean	6384.47	27.92 ± 0.75	0.07
Standard Error	476.08	2.44 ± 0.21	
CV^2	0.028		
CE^2	0.005		
CE^2 / CV^2	0.177		

CVB^2	0.023		
CVB^2 (% of CV^2)	82.33%		
**HDSD / 6M / Impoverished Environment**			
**Subjects**	**N**	**Thickness (μm)**	**CE (Scheaffer)**
HDSD 6M IE Animal 1	7452.72	23.21 ± 1.03	0.05
HDSD 6M IE Animal 2	5296.34	19.63 ± 1.12	0.06
HDSD 6M IE Animal 3	8461.34	25.32 ± 0.85	0.05
HDSD 6M IE Animal 4	7997.01	21.85 ± 1.44	0.05
HDSD 6M IE Animal 5	7149.91	26.08 ± 1.12	0.06
Mean	7271.46	23.51 ± 0.71	0.05
Standard Error	542.72	1.17 ± 0.1	
CV^2	0.028		
CE^2	0.003		
CE^2 / CV^2	0.108		
CVB^2	0.025		
CVB^2 (% of CV^2)	89.22%		
**HDSDHD / 6M / Impoverished Environment**			
**Subjects**	**N**	**Thickness (μm)**	**CE (Scheaffer)**
HDSDHD 6M IE Animal 1	9259.55	21.24 ± 0.95	0.05
HDSDHD 6M IE Animal 2	18258.08	25.17 ± 0.73	0.04
HDSDHD 6M IE Animal 3	5498.17	14.72 ± 0.6	0.05
HDSDHD 6M IE Animal 4	8258.57	24.63 ± 0.93	0.05
HDSDHD 6M IE Animal 5	4982.95	16.95 ± 0.94	0.06
Mean	9251.46	20.54 ± 0.83	0.05
Standard Error	2391.78	2.07 ± 0.07	
CV^2	0.334		

CE^2	0.003		
CE^2 / CV^2	0.008		
CVB^2	0.332		
CVB^2 (% of CV^2)	99.24%		
**HD / 18M / Impoverished Environment**			
**Subjects**	**N**	**Thickness (µm)**	**CE (Scheaffer)**
HD 18M IE Animal 1	15423.60	24.53 ± 0.43	0.04
HD 18M IE Animal 2	6012.99	26.29 ± 0.14	0.06
HD 18M IE Animal 3	6774.91	21.44 ± 1.62	0.06
HD 18M IE Animal 4	13415.09	22.85 ± 0.08	0.04
HD 18M IE Animal 5	6732.19	20.1 ± 1.7	0.06
Mean	9671.76	23.04 ± 0.79	0.05
Standard Error	1968.70	1.1 ± 0.36	
CV^2	0.207		
CE^2	0.003		
CE^2 / CV^2	0.013		
CVB^2	0.205		
CVB^2 (% of CV^2)	98.75%		
**HDSD / 18M / Impoverished Environment**			
**Subjects**	**N**	**Thickness (µm)**	**CE (Scheaffer)**
HDSD 18M IE Animal 1	7451.57	20.32 ± 0.47	0.04
HDSD 18M IE Animal 2	13070.89	21.17 ± 1.18	0.04
HDSD 18M IE Animal 3	5378.97	16.28 ± 1.23	0.06
HDSD 18M IE Animal 4	12510.54	22.48 ± 0.18	0.05
HDSD 18M IE Animal 5	13125.94	25.59 ± 0.52	0.05
Mean	10307.58	21.17 ± 0.72	0.05

Standard Error	1626.04	1.52 ± 0.21	
CV^2	0.124		
CE^2	0.002		
CE^2 / CV^2	0.018		
CVB^2	0.122		
CVB^2 (% of CV^2)	98.17%		
**HDSDHD / 18M / Impoverished Environment**			
**Subjects**	**N**	**Thickness (μm)**	**CE (Scheaffer)**
HDSDHD 18M IE Animal 1	16206.96	26.07 ± 0.9	0.04
HDSDHD 18M IE Animal 2	14815.85	26.28 ± 0.42	0.04
HDSDHD 18M IE Animal 3	4638.35	14.51 ± 0.49	0.06
HDSDHD 18M IE Animal 4	12920.49	22.68 ± 0.19	0.05
HDSDHD 18M IE Animal 5	5780.36	20.01 ± 1.35	0.06
Mean	10872.40	21.91 ± 0.67	0.05
Standard Error	2376.92	2.18 ± 0.21	
CV^2	0.239		
CE^2	0.002		
CE^2 / CV^2	0.010		
CVB^2	0.237		
CVB^2 (% of CV^2)	98.99%		
**HD / 6M / Enriched Environment**			
**Subjects**	**N**	**Thickness (μm)**	**CE (Scheaffer)**
HD 6M EE Animal 1	10657.77	21.73 ± 1	0.05
HD 6M EE Animal 2	7652.81	21.69 ± 0.2	0.06
HD 6M EE Animal 3	4886.57	22.08 ± 0.74	0.07
HD 6M EE Animal 4	9336.43	22.08 ± 0.74	0.04
HD 6M EE Animal 5	9162.46	18.57 ± 0.93	0.05
Mean	8339.21	20.95 ± 0.68	0.05
Standard Error	985.89	0.64 ± 0.14	

CV^2	0.054		
CE^2	0.003		
CE^2 / CV^2	0.050		
CVB^2	0.052		
CVB^2 (% of CV^2)	95.01%		
**HDSD / 6M / Enriched Environment**			
**Subjects**	**N**	**Thickness (µm)**	**CE (Scheaffer)**
HDSD 6M EE Animal 1	7573.13	24.54 ± 0.51	0.05
HDSD 6M EE Animal 2	7687.63	18.17 ± 1.77	0.05
HDSD 6M EE Animal 3	7360.45	22.24 ± 0.25	0.07
HDSD 6M EE Animal 4	7478.44	22.65 ± 1.23	0.06
Mean	7524.91	21.90 ± 0.94	0.06
Standard Error	69.53	1.34 ± 0.35	
CV^2	0.0003		
CE^2	0.003		
CE^2 / CV^2	9.249		
CVB^2	-0.003		
CVB^2 (% of CV^2)	-824.87%		
**HDSDHD / 6M / Enriched Environment**			
**Subjects**	**N**	**Thickness (µm)**	**CE (Scheaffer)**
HDSDHD 6M EE Animal 1	8246.25	24.42 ± 0.59	0.05
HDSDHD 6M EE Animal 2	7720.45	19.73 ± 1.62	0.05
HDSDHD 6M EE Animal 3	6861.96	18.03 ± 0.98	0.06
HDSDHD 6M EE Animal 4	8391.70	22.7 ± 0.09	0.06
HDSDHD 6M EE Animal 5	6701.12	22.3 ± 1.21	0.05
Mean	7584.29	21.43 ± 0.9	0.05
Standard Error	347.16	1.13 ± 0.26	
CV^2	2.479		
CE^2	0.003		

CE^2 / CV^2	0.001		
CVB^2	2.476		
CVB^2 (% of CV^2)	99.88%		
**HD / 18M / Enriched Environment**			
**Subjects**	**N**	**Thickness (µm)**	**CE (Scheaffer)**
HD 18M EE Animal 1	6193.39	23.99 ± 0.16	0.06
HD 18M EE Animal 2	10397.41	22.3 ± 0.45	0.05
HD 18M EE Animal 3	8002.37	19.9 ± 1.42	0.06
HD 18M EE Animal 4	12435.54	22.53 ± 0.09	0.05
HD 18M EE Animal 5	5634.91	18.78 ± 1.31	0.06
Mean	8532.72	21.5 ± 0.69	0.05
Standard Error	1281.80	0.95 ± 0.28	
CV^2	0.113		
CE^2	0.003		
CE^2 / CV^2	0.026		
CVB^2	0.110		
CVB^2 (% of CV^2)	97.38%		
**HDSD / 18M / Enriched Environment**			
**Subjects**	**N**	**Thickness (µm)**	**CE (Scheaffer)**
HDSD 18M EE Animal 1	10088.97	23.23 ± 0.17	0.04
HDSD 18M EE Animal 2	10327.10	23.01 ± 0.78	0.05
HDSD 18M EE Animal 3	6681.43	16.9 ± 0.35	0.06
HDSD 18M EE Animal 4	10075.85	22.75 ± 0.13	0.05
HDSD 18M EE Animal 5	5662.37	15.29 ± 0.54	0.05
Mean	8567.14	20.24 ± 0.39	0.05
Standard Error	992.05	1.71 ± 0.12	
CV^2	0.067		
CE^2	0.003		
CE^2 / CV^2	0.038		

CVB^2	0.064		
CVB^2 (% of CV^2)	96.15%		
**HDSDHD / 18M / Enriched Environment**			
**Subjects**	**N**	**Thickness (µm)**	**CE (Scheaffer)**
HDSDHD 18M EE Animal 1	10841.92	22.89 ± 0.27	0.04
HDSDHD 18M EE Animal 2	8676.16	21.61 ± 0.42	0.05
HDSDHD 18M EE Animal 3	9768.88	17.87 ± 0.87	0.05
HDSDHD 18M EE Animal 4	14854.96	22.7 ± 0.05	0.05
HDSDHD 18M EE Animal 5	7962.72	22.84 ± 0.7	0.06
Mean	10420.93	21.58 ± 0.46	0.05
Standard Error	1211.40	0.96 ± 0.15	
CV^2	0.068		
CE^2	0.002		
CE^2 / CV^2	0.033		
CVB^2	0.065		
CVB^2 (% of CV^2)	96.70%		

$CVB^2 = CV^2 - CE^2$ (CV, coefficient of variation; CVB, biological coefficient of variation; CE, coefficient of error). N = number of astrocytes; Mean = mean numbers in each group; 6M and 18M indicate 6 months old and 18 months old, respectively.

Table S5. Estimated Unilateral Numbers of Astrocytes (N) With the Coefficient of Error (CE) for the Stratum Radiatum of CA3 of 6-, and 18-Month-Old Female

Albino Swiss Mice Fed a Hard Diet (HD), Hard/Soft Diet (HD/SD) and Hard/Soft/Hard Diet (HDSDHD).

STRATUM RADIATUM – CA3			
HD / 6M / Impoverished Environment			
Subjects	N	Thickness (μm)	CE (Scheaffer)
HD 6M IE Animal 1	14593.03	30.18 ± 1.09	0.04
HD 6M IE Animal 2	8010.94	24.11 ± 0.48	0.05
HD 6M IE Animal 3	16166.66	30.41 ± 1.07	0.04
HD 6M IE Animal 4	15122.66	33.65 ± 1.24	0.04
HD 6M IE Animal 5	9498.77	25.75 ± 0.69	0.05
Mean	12678.41	28.82 ± 0.92	0.04
Standard Error	1638.65	1.72 ± 0.14	
CV^2	0.084		
CE^2	0.002		
CE^2 / CV^2	0.020		
CVB^2	0.082		
CVB^2 (% of CV^2)	97.98%		
HDSD / 6M / Impoverished Environment			
Subjects	N	Thickness (μm)	CE (Scheaffer)
HDSD 6M IE Animal 1	11499.17	25.43 ± 0.89	0.04
HDSD 6M IE Animal 2	9963.00	25.18 ± 2	0.05
HDSD 6M IE Animal 3	13290.43	26.17 ± 0.45	0.04
HDSD 6M IE Animal 4	12658.97	21.61 ± 1.66	0.04
HDSD 6M IE Animal 5	13831.29	26.24 ± 0.39	0.04

Mean	12248.57	24.93 ± 1.08	0.04
Standard Error	690.81	0.85 ± 0.32	
CV^2	0.016		
CE^2	0.002		
CE^2 / CV^2	0.110		
CVB^2	0.014		
CVB^2 (% of CV^2)	89.01%		
**HDSDHD / 6M / Impoverished Environment**			
**Subjects**	**N**	**Thickness (µm)**	**CE (Scheaffer)**
HDSDHD 6M IE Animal 1	13521.86	25.03 ± 1.64	0.03
HDSDHD 6M IE Animal 2	6048.51	25.96 ± 0.37	0.05
HDSDHD 6M IE Animal 3	13428.26	21.03 ± 0.42	0.03
HDSDHD 6M IE Animal 4	10104.51	25.02 ± 0.96	0.04
HDSDHD 6M IE Animal 5	15075.00	20.16 ± 0.92	0.03
Mean	11635.63	23.44 ± 0.86	0.04
Standard Error	1614.94	1.18 ± 0.23	
CV^2	0.096		
CE^2	0.001		
CE^2 / CV^2	0.015		
CVB^2	0.095		
CVB^2 (% of CV^2)	98.55%		
**HD / 18M / Impoverished Environment**			
**Subjects**	**N**	**Thickness (µm)**	**CE (Scheaffer)**
HD 18M IE Animal 1	18671.91	24.5 ± 0.57	0.04
HD 18M IE Animal 2	9399.77	26.28 ± 0.26	0.04
HD 18M IE Animal 3	11780.57	24.37 ± 1.15	0.05
HD 18M IE Animal 4	17547.00	22.75 ± 0.08	0.04

HD 18M IE Animal 5	16538.40	22.28 ± 0.9	0.04
Mean	14787.53	24.04 ± 0.59	0.04
Standard Error	1786.59	0.71 ± 0.2	
CV^2	0.073		
CE^2	0.002		
CE^2 / CV^2	0.023		
CVB^2	0.071		
CVB^2 (% of CV^2)	97.73%		
**HDSD / 18M / Impoverished Environment**			
**Subjects**	**N**	**Thickness (µm)**	**CE (Scheaffer)**
HDSD 18M IE Animal 1	12113.40	20.53 ± 0.77	0.04
HDSD 18M IE Animal 2	17137.89	21.98 ± 1.05	0.04
HDSD 18M IE Animal 3	10817.66	16.56 ± 0.92	0.04
HDSD 18M IE Animal 4	13453.29	22.62 ± 0.09	0.04
HDSD 18M IE Animal 5	32735.40	25.96 ± 0.53	0.04
Mean	17251.53	21.53 ± 0.67	0.04
Standard Error	4012.39	1.53 ± 0.17	
CV^2	0.270		
CE^2	0.001		
CE^2 / CV^2	0.006		
CVB^2	0.269		
CVB^2 (% of CV^2)	99.45%		
**HDSDHD / 18M / Impoverished Environment**			
**Subjects**	**N**	**Thickness (µm)**	**CE (Scheaffer)**
HDSDHD 18M IE Animal 1	22165.11	26.86 ± 0.78	0.04
HDSDHD 18M IE Animal 2	12348.77	23.89 ± 0.6	0.04
HDSDHD 18M IE Animal 3	8292.94	13.68 ± 0.44	0.04
HDSDHD 18M IE Animal 4	9421.71	22.9 ± 0.16	0.05

HDSDHD 18M IE Animal 5	9375.77	20.56 ± 1.07	0.05
Mean	12320.86	21.58 ± 0.61	0.04
Standard Error	2551.60	2.22 ± 0.15	
CV^2	0.214		
CE^2	0.002		
CE^2 / CV^2	0.008		
CVB^2	0.213		
CVB^2 (% of CV^2)	99.17%		
**HD / 6M / Enriched Environment**			
**Subjects**	**N**	**Thickness (µm)**	**CE (Scheaffer)**
HD 6M EE Animal 1	13491.26	20.53 ± 0.62	0.04
HD 6M EE Animal 2	10096.54	21.95 ± 0.39	0.04
HD 6M EE Animal 3	4886.57	21.71 ± 0.9	0.06
HD 6M EE Animal 4	10923.34	18.53 ± 0.81	0.04
HD 6M EE Animal 5	11980.89	20.24 ± 0.74	0.04
Mean	10275.72	20.59 ± 0.69	0.04
Standard Error	1461.88	0.61 ± 0.09	
CV^2	4.316		
CE^2	0.002		
CE^2 / CV^2	0.000		
CVB^2	4.315		
CVB^2 (% of CV^2)	99.96%		
**HDSD / 6M / Enriched Environment**			
**Subjects**	**N**	**Thickness (µm)**	**CE (Scheaffer)**
HDSD 6M EE Animal 1	4758.94	22.28 ± 0.33	0.06
HDSD 6M EE Animal 2	9632.91	17.83 ± 1.5	0.04
HDSD 6M EE Animal 3	8757.34	22.07 ± 0.34	0.05
HDSD 6M EE Animal 4	6431.83	19 ± 0.97	0.06
Mean	7395.26	20.3 ± 0.78	0.05

Standard Error	1108.33	1.11 ± 0.28	
CV^2	0.0898		
CE^2	0.003		
CE^2 / CV^2	0.032		
CVB^2	0.087		
CVB^2 (% of CV^2)	96.85%		
**HDSDHD / 6M / Enriched Environment**			
**Subjects**	**N**	**Thickness (μm)**	**CE (Scheaffer)**
HDSDHD 6M EE Animal 1	12212.23	22.63 ± 0.49	0.04
HDSDHD 6M EE Animal 2	7551.60	18.99 ± 1.08	0.05
HDSDHD 6M EE Animal 3	10336.80	18.69 ± 0.87	0.05
HDSDHD 6M EE Animal 4	11319.00	22.67 ± 0.06	0.04
HDSDHD 6M EE Animal 5	7357.54	22.27 ± 0.97	0.06
Mean	9755.43	21.05 ± 0.69	0.05
Standard Error	985.53	0.91 ± 0.19	
CV^2	0.051		
CE^2	0.002		
CE^2 / CV^2	0.043		
CVB^2	0.049		
CVB^2 (% of CV^2)	95.69%		
**HD / 18M / Enriched Environment**			
**Subjects**	**N**	**Thickness (μm)**	**CE (Scheaffer)**
HD 18M EE Animal 1	10575.26	23.65 ± 0.24	0.04
HD 18M EE Animal 2	12901.03	21.89 ± 0.28	0.04
HD 18M EE Animal 3	12434.57	19.99 ± 0.94	0.05
HD 18M EE Animal 4	12488.23	22.59 ± 0.11	0.04

HD 18M EE Animal 5	7176.60	18.69 ± 0.53	0.05
Mean	11115.14	21.36 ± 0.42	0.04
Standard Error	1063.47	0.9 ± 0.15	
CV^2	0.046		
CE^2	0.002		
CE^2 / CV^2	0.043		
CVB^2	0.044		
CVB^2 (% of CV^2)	95.73%		
**HDSD / 18M / Enriched Environment**			
**Subjects**	**N**	**Thickness (µm)**	**CE (Scheaffer)**
HDSD 18M EE Animal 1	12905.06	22.81 ± 0.23	0.04
HDSD 18M EE Animal 2	14964.86	23.42 ± 0.83	0.04
HDSD 18M EE Animal 3	13116.26	18.25 ± 0.76	0.04
HDSD 18M EE Animal 4	9376.37	22.81 ± 0.05	0.05
HDSD 18M EE Animal 5	6234.60	15.41 ± 0.36	0.05
Mean	11319.43	20.54 ± 0.45	0.04
Standard Error	1559.85	1.58 ± 0.15	
CV^2	0.095		
CE^2	0.002		
CE^2 / CV^2	0.020		
CVB^2	0.093		
CVB^2 (% of CV^2)	98.03%		
**HDSDHD / 18M / Enriched Environment**			
**Subjects**	**N**	**Thickness (µm)**	**CE (Scheaffer)**
HDSDHD 18M EE Animal 1	10925.74	23.44 ± 0.31	0.04
HDSDHD 18M EE Animal 2	13150.89	21.7 ± 0.4	0.04
HDSDHD 18M EE Animal 3	11238.00	17.67 ± 0.59	0.04

HDSDHD 18M EE Animal 4	17173.20	22.79 ± 0.04	0.05
HDSDHD 18M EE Animal 5	10076.66	22.17 ± 0.57	0.04
Mean	12512.90	21.55 ± 0.38	0.04
Standard Error	1268.96	1.01 ± 0.1	
CV^2	0.051		
CE^2	0.002		
CE^2 / CV^2	0.035		
CVB^2	0.050		
CVB^2 (% of CV^2)	96.52%		

$CVB^2 = CV^2 - CE^2$ (CV, coefficient of variation; CVB, biological coefficient of variation; CE, coefficient of error). N = number of astrocytes; Mean = mean numbers in each group; 6M and 18M indicate 6 months old and 18 months old, respectively.

Table S6. Estimated Unilateral Numbers of Astrocytes (N) With the Coefficient of Error (CE) for the Stratum Oriens of CA3 of 6-, and 18-Month-Old Female Albino Swiss Mice Fed a Hard Diet (HD), Hard/Soft Diet (HD/SD) and Hard/Soft/Hard Diet (HDSDHD).

**STRATUM ORIENS – CA3**			
**HD / 6M / Impoverished Environment**			
**Subjects**	**N**	**Thickness (µm)**	**CE (Scheaffer)**
HD 6M IE Animal 1	16145.74	28.73 ± 0.85	0.04

HD 6M IE Animal 2	12813.51	24.94 ± 0.71	0.04
HD 6M IE Animal 3	31023.94	32.16 ± 0.76	0.03
HD 6M IE Animal 4	22152.94	33.81 ± 1.32	0.03
HD 6M IE Animal 5	13489.89	26.26 ± 0.55	0.04
Mean	19125.21	29.18 ± 0.84	0.04
Standard Error	3400.14	1.69 ± 0.13	
CV^2	0.158		
CE^2	0.001		
CE^2 / CV^2	0.009		
CVB^2	0.157		
CVB^2 (% of CV^2)	99.12%		
**HDSD / 6M / Impoverished Environment**			
**Subjects**	**N**	**Thickness (µm)**	**CE (Scheaffer)**
HDSD 6M IE Animal 1	13479.26	25.87 ± 0.79	0.04
HDSD 6M IE Animal 2	8900.06	27.1 ± 3.01	0.05
HDSD 6M IE Animal 3	14658.94	26.24 ± 0.38	0.04
HDSD 6M IE Animal 4	16866.09	23.29 ± 1.12	0.04
HDSD 6M IE Animal 5	15481.71	27.21 ± 0.39	0.04
Mean	13877.21	25.94 ± 1.14	0.04
Standard Error	1361.05	0.71 ± 0.49	
CV^2	0.048		
CE^2	0.002		
CE^2 / CV^2	0.037		
CVB^2	0.046		
CVB^2 (% of CV^2)	96.35%		
**HDSDHD / 6M / Impoverished Environment**			
**Subjects**	**N**	**Thickness (µm)**	**CE (Scheaffer)**

HDSDHD 6M IE Animal 1	18115.71	26.15 ± 1.2	0.03
HDSDHD 6M IE Animal 2	14394.94	26.19 ± 0.48	0.04
HDSDHD 6M IE Animal 3	15123.86	22.31 ± 0.62	0.04
HDSDHD 6M IE Animal 4	16029.86	25.38 ± 0.9	0.04
HDSDHD 6M IE Animal 5	15274.89	22.11 ± 1.41	0.03
Mean	15787.85	24.43 ± 0.92	0.04
Standard Error	637.25	0.92 ± 0.17	
CV^2	0.008		
CE^2	0.001		
CE^2 / CV^2	0.159		
CVB^2	0.007		
CVB^2 (% of CV^2)	84.09%		
**HD / 18M / Impoverished Environment**			
**Subjects**	**N**	**Thickness (μm)**	**CE (Scheaffer)**
HD 18M IE Animal 1	14229.08	25.2 ± 0.91	0.04
HD 18M IE Animal 2	11626.37	26.49 ± 0.16	0.04
HD 18M IE Animal 3	14641.39	25.97 ± 0.76	0.04
HD 18M IE Animal 4	19573.46	22.76 ± 0.13	0.04
HD 18M IE Animal 5	16992.17	24.13 ± 0.96	0.04
Mean	15412.49	24.91 ± 0.58	0.04
Standard Error	1344.22	0.67 ± 0.18	
CV^2	0.038		
CE^2	0.002		
CE^2 / CV^2	0.044		
CVB^2	0.036		
CVB^2 (% of CV^2)	95.56%		
**HDSD / 18M / Impoverished Environment**			
**Subjects**	**N**	**Thickness (μm)**	**CE (Scheaffer)**

HDSD 18M IE Animal 1	17410.02	21.72 ± 0.89	0.04
HDSD 18M IE Animal 2	18946.89	23.37 ± 1.33	0.04
HDSD 18M IE Animal 3	15305.14	19.01 ± 0.45	0.04
HDSD 18M IE Animal 4	16827.09	22.66 ± 0.13	0.04
HDSD 18M IE Animal 5	35493.86	28.62 ± 0.61	0.03
Mean	20796.60	23.08 ± 0.68	0.04
Standard Error	3720.30	1.57 ± 0.2	
CV^2	0.160		
CE^2	0.001		
CE^2 / CV^2	0.009		
CVB^2	0.159		
CVB^2 (% of CV^2)	99.11%		
**HDSDHD / 18M / Impoverished Environment**			
**Subjects**	**N**	**Thickness (µm)**	**CE (Scheaffer)**
HDSDHD 18M IE Animal 1	23555.91	28.05 ± 0.75	0.04
HDSDHD 18M IE Animal 2	14862.29	26.03 ± 0.57	0.03
HDSDHD 18M IE Animal 3	12602.23	15.89 ± 0.76	0.04
HDSDHD 18M IE Animal 4	11479.71	22.84 ± 0.15	0.05
HDSDHD 18M IE Animal 5	12617.91	24.17 ± 1.65	0.05
Mean	15023.61	23.4 ± 0.77	0.04
Standard Error	2202.68	2.07 ± 0.24	
CV^2	0.107		
CE^2	0.002		
CE^2 / CV^2	0.016		
CVB^2	0.106		
CVB^2 (% of CV^2)	98.44%		
**HD / 6M / Enriched Environment**			

Subjects	N	Thickness (µm)	CE (Scheaffer)
HD 6M EE Animal 1	15015.77	21.39 ± 0.75	0.04
HD 6M EE Animal 2	8134.37	22.07 ± 0.22	0.05
HD 6M EE Animal 3	8214.43	22.02 ± 0.87	0.04
HD 6M EE Animal 4	13109.49	20.03 ± 0.35	0.04
HD 6M EE Animal 5	14859.00	23.35 ± 1.01	0.04
Mean	11866.61	21.77 ± 0.64	0.04
Standard Error	1544.09	0.54 ± 0.15	
CV^2	0.085		
CE^2	0.002		
CE^2 / CV^2	0.022		
CVB^2	0.083		
CVB^2 (% of CV^2)	97.84%		
**HDSD / 6M / Enriched Environment**			
**Subjects**	**N**	**Thickness (µm)**	**CE (Scheaffer)**
HDSD 6M EE Animal 1	11086.46	24.16 ± 0.19	0.04
HDSD 6M EE Animal 2	12603.34	18.87 ± 1.38	0.04
HDSD 6M EE Animal 3	12684.00	21.83 ± 0.3	0.05
HDSD 6M EE Animal 4	8575.20	24.15 ± 0.78	0.05
Mean	11237.25	22.25 ± 0.66	0.05
Standard Error	960.41	1.25 ± 0.27	
CV^2	0.0292		
CE^2	0.002		
CE^2 / CV^2	0.073		
CVB^2	0.027		
CVB^2 (% of CV^2)	92.66%		
**HDSDHD / 6M / Enriched Environment**			

Subjects	N	Thickness (µm)	CE (Scheaffer)
HDSDHD 6M EE Animal 1	15315.94	23.76 ± 0.59	0.04
HDSDHD 6M EE Animal 2	15102.69	20.54 ± 0.38	0.04
HDSDHD 6M EE Animal 3	12304.89	19.86 ± 1.11	0.05
HDSDHD 6M EE Animal 4	12158.23	22.67 ± 0.04	0.05
HDSDHD 6M EE Animal 5	11663.14	25.69 ± 0.47	0.05
Mean	13308.98	22.5 ± 0.52	0.04
Standard Error	783.79	1.06 ± 0.17	
CV^2	0.017		
CE^2	0.002		
CE^2 / CV^2	0.116		
CVB^2	0.015		
CVB^2 (% of CV^2)	88.43%		
**HD / 18M / Enriched Environment**			
**Subjects**	**N**	**Thickness (µm)**	**CE (Scheaffer)**
HD 18M EE Animal 1	10575.26	23.65 ± 0.24	0.04
HD 18M EE Animal 2	12901.03	21.89 ± 0.28	0.04
HD 18M EE Animal 3	12434.57	19.99 ± 0.94	0.05
HD 18M EE Animal 4	12488.23	22.59 ± 0.11	0.04
HD 18M EE Animal 5	7176.60	18.69 ± 0.53	0.05
Mean	11115.14	21.36 ± 0.42	0.04
Standard Error	1063.47	0.9 ± 0.15	
CV^2	0.046		
CE^2	0.002		
CE^2 / CV^2	0.043		
CVB^2	0.044		
CVB^2 (% of CV^2)	95.73%		

HDSD / 18M / Enriched Environment			
**Subjects**	**N**	**Thickness (µm)**	**CE (Scheaffer)**
HDSD 18M EE Animal 1	8565.51	23.69 ± 0.12	0.05
HDSD 18M EE Animal 2	18323.40	23.85 ± 0.3	0.04
HDSD 18M EE Animal 3	13679.49	20.95 ± 1.04	0.05
HDSD 18M EE Animal 4	10696.80	22.57 ± 0.1	0.05
HDSD 18M EE Animal 5	9811.89	23.94 ± 1.55	0.05
Mean	12215.42	23 ± 0.62	0.05
Standard Error	1744.36	0.57 ± 0.29	
CV^2	0.102		
CE^2	0.002		
CE^2 / CV^2	0.020		
CVB^2	0.100		
CVB^2 (% of CV^2)	97.96%		

HDSDHD / 18M / Enriched Environment			
**Subjects**	**N**	**Thickness (µm)**	**CE (Scheaffer)**
HDSDHD 18M EE Animal 1	17229.69	22.83 ± 0.19	0.04
HDSDHD 18M EE Animal 2	16386.00	24.73 ± 0.69	0.04
HDSDHD 18M EE Animal 3	13125.94	19.08 ± 0.47	0.04
HDSDHD 18M EE Animal 4	11479.46	22.71 ± 0.13	0.05
HDSDHD 18M EE Animal 5	9487.29	20.66 ± 0.8	0.04
Mean	13541.67	22 ± 0.45	0.04
Standard Error	1458.69	0.97 ± 0.13	
CV^2	0.058		
CE^2	0.002		
CE^2 / CV^2	0.031		
CVB^2	0.056		
CVB^2 (% of CV^2)	96.94%		

$CVB^2 = CV^2 - CE^2$ (CV, coefficient of variation; CVB, biological coefficient of variation; CE, coefficient of error). N = number of astrocytes; Mean = mean numbers in each group; 6M and 18M indicate 6 months old and 18 months old, respectively.

Table S7. Estimated Unilateral Numbers of Astrocytes (N) With the Coefficient of Error (CE) for the Stratum Moleculare of Dentate Gyrus of 6-, and 18-Month-Old Female Albino Swiss Mice Fed a Hard Diet (HD), Hard/Soft Diet (HD/SD) and Hard/Soft/Hard Diet (HD/SD/HD).

STRATUM MOLECULARE – DENTATE GYRUS			
**HD / 6M / Impoverished Environment**			
**Subjects**	**N**	**Thickness (µm)**	**CE (Scheaffer)**
HD 6M IE Animal 1	32762.48	26.2 ± 0.13	0.03
HD 6M IE Animal 2	34637.74	26.5 ± 0.05	0.03
HD 6M IE Animal 3	24791.74	24.8 ± 0.2	0.04
HD 6M IE Animal 4	26607.43	26 ± 0.18	0.03
HD 6M IE Animal 5	32381.14	25.31 ± 0.16	0.03
Mean	30236.11	25.77 ± 0.15	0.03
Standard Error	1912.67	0.31 ± 0.02	
CV^2	0.02		
CE^2	0.001		
CE^2 / CV^2	0.05		
CVB^2	0.02		
CVB^2 (% of CV^2)	94.93%		
**HDSD / 6M / Impoverished Environment**			
**Subjects**	**N**	**Thickness (µm)**	**CE (Scheaffer)**
HDSD 6M IE Animal 1	24271.85	25.75 ± 0.81	0.04
HDSD 6M IE Animal 2	29155	23.12 ± 0.98	0.03
HDSD 6M IE Animal 3	36534.17	25.37 ± 0.28	0.03
HDSD 6M IE Animal 4	28660.03	23.52 ± 0.29	0.03
HDSD 6M IE Animal 5	21163	22.46 ± 0.5	0.03

Mean	27956.81	24.05 ± 0.59	0.03
Standard Error	2600.35	0.64 ± 0.16	
CV^2	0.043		
CE^2	0.001		
CE^2 / CV^2	0.001		
CVB^2	0.042		
CVB^2 (% of CV^2)	97.64%		
**HDSDHD / 6M / Impoverished Environment**			
**Subjects**	**N**	**Thickness (μm)**	**CE (Scheaffer)**
HDSDHD 6M IE Animal 1	25457.48	24.2 ± 0.18	0.03
HDSDHD 6M IE Animal 2	31548.09	25.01 ± 0.14	0.03
HDSDHD 6M IE Animal 3	43042.29	24.07 ± 0.39	0.03
HDSDHD 6M IE Animal 4	25881.09	22.4 ± 0.4	0.03
HDSDHD 6M IE Animal 5	31381.71	22.9 ± 0.49	0.03
Mean	31462.13	23.72 ± 0.32	0.03
Standard Error	3172.68	0.47 ± 0.07	
CV^2	0.051		
CE^2	0.001		
CE^2 / CV^2	0.016		
CVB^2	0.05		
CVB^2 (% of CV^2)	98.36%		
**HD / 18M / Impoverished Environment**			
**Subjects**	**N**	**Thickness (μm)**	**CE (Scheaffer)**
HD 18M IE Animal 1	36240.17	25.5 ± 0.21	0.03
HD 18M IE Animal 2	18044.31	24.97 ± 0.32	0.04
HD 18M IE Animal 3	22563.94	25.18 ± 0.31	0.04
HD 18M IE Animal 4	26491.03	25.02 ± 0.75	0.03
HD 18M IE Animal 5	32960.91	25.56 ± 0.44	0.03
Mean	27260.07	25.25 ± 0.41	0.03
Standard Error	3322.02	0.12 ± 0.09	
CV^2	0.074		
CE^2	0.001		
CE^2 / CV^2	0.015		
CVB^2	0.073		

CVB^2 (% of CV^2)	98.51%		
**HDSD / 18M / Impoverished Environment**			
**Subjects**	**N**	**Thickness (µm)**	**CE (Scheaffer)**
HDSD 18M IE Animal 1	24294.69	23.69 ± 0.71	0.03
HDSD 18M IE Animal 2	22587.94	25.3 ± 0.73	0.04
HDSD 18M IE Animal 3	27544.03	24.98 ± 0.5	0.03
HDSD 18M IE Animal 4	10749.43	23.87 ± 0.53	0.05
HDSD 18M IE Animal 5	21564.51	27.02 ± 0.25	0.03
Mean	21348.12	24.97 ± 0.55	0.04
Standard Error	2837.14	0.6 ± 0.09	
CV^2	0.088		
CE^2	0.001		
CE^2 / CV^2	0.016		
CVB^2	0.087		
CVB^2 (% of CV^2)	98.41%		
**HDSDHD / 18M / Impoverished Environment**			
**Subjects**	**N**	**Thickness (µm)**	**CE (Scheaffer)**
HDSDHD 18M IE Animal 1	18373.89	26.05 ± 0.18	0.04
HDSDHD 18M IE Animal 2	22414.29	24 ± 0.64	0.03
HDSDHD 18M IE Animal 3	31035.69	22.39 ± 0.35	0.03
HDSDHD 18M IE Animal 4	20105.74	24.78 ± 0.45	0.04
HDSDHD 18M IE Animal 5	21732.69	23.38 ± 1.01	0.04
Mean	22732.46	24.12 ± 0.52	0.03
Standard Error	2190.22	0.62 ± 0.14	
CV^2	0.046		
CE^2	0.001		
CE^2 / CV^2	0.026		
CVB^2	0.045		
CVB^2 (% of CV^2)	97.42%		
**HD / 6M / Enriched Environment**			
**Subjects**	**N**	**Thickness (µm)**	**CE (Scheaffer)**
HD 6M EE Animal 1	23997	24.31 ± 0.4	0.03
HD 6M EE Animal 2	27170.66	21.39 ± 0.38	0.03
HD 6M EE Animal 3	20119.8	21.64 ± 1.22	0.04

HD 6M EE Animal 4	31987.46	23.1 ± 0.37	0.03
HD 6M EE Animal 5	30681.94	24.5 ± 0.32	0.03
Mean	26791.37	22.99 ± 0.54	0.03
Standard Error	2174.83	0.65 ± 0.17	
CV^2	0.033		
CE^2	0.001		
CE^2 / CV^2	0.032		
CVB^2	0.032		
CVB^2 (% of CV^2)	96.79%		
**HDSD / 6M / Enriched Environment**			
**Subjects**	**N**	**Thickness (µm)**	**CE (Scheaffer)**
HDSD 6M EE Animal 1	14059.71	19.14 ± 0.84	0.04
HDSD 6M EE Animal 2	22781.31	20.27 ± 0.67	0.04
HDSD 6M EE Animal 3	56766.26	20.53 ± 0.33	0.02
HDSD 6M EE Animal 4	36989.57	19.64 ± 0.37	0.03
HDSD 6M EE Animal 5	24346.11	20.55 ± 0.73	0.03
Mean	30988.59	20.03 ± 0.59	0.03
Standard Error	7411.37	0.28 ± 0.1	
CV^2	0.286		
CE^2	0.001		
CE^2 / CV^2	0.004		
CVB^2	0.285		
CVB^2 (% of CV^2)	99.64%		
**HDSDHD / 6M / Enriched Environment**			
**Subjects**	**N**	**Thickness (µm)**	**CE (Scheaffer)**
HDSDHD 6M EE Animal 1	34876.2	17.95 ± 0.85	0.03
HDSDHD 6M EE Animal 2	18141.26	17.91 ± 0.93	0.04
HDSDHD 6M EE Animal 3	16736.31	20.77 ± 0.72	0.04
HDSDHD 6M EE Animal 4	31279.11	20.75 ± 0.46	0.03
HDSDHD 6M EE Animal 5	29055.34	24.84 ± 0.61	0.03
Mean	26017.65	20.45 ± 0.71	0.03
Standard Error	3630.19	1.27 ± 0.08	
CV^2	0.097		
CE^2	0.001		

CE^2 / CV^2	0.011		
CVB^2	0.096		
CVB^2 (% of CV^2)	98.94%		
**HD / 18M / Enriched Environment**			
**Subjects**	**N**	**Thickness (μm)**	**CE (Scheaffer)**
HD 18M EE Animal 1	22571.91	20.44 ± 0.94	0.04
HD 18M EE Animal 2	27194.4	22.16 ± 1.49	0.03
HD 18M EE Animal 3	24906.26	21.48 ± 0.76	0.03
HD 18M EE Animal 4	30110.91	21.19 ± 0.15	0.03
HD 18M EE Animal 5	19483.71	24.34 ± 0.62	0.04
Mean	24853.44	21.92 ± 0.79	0.03
Standard Error	1832.53	0.67 ± 0.22	
CV^2	0.027		
CE^2	0.001		
CE^2 / CV^2	0.043		
CVB^2	0.026		
CVB^2 (% of CV^2)	95.65%		
**HDSD / 18M / Enriched Environment**			
**Subjects**	**N**	**Thickness (μm)**	**CE (Scheaffer)**
HDSD 18M EE Animal 1	28600.29	19.58 ± 0.22	0.03
HDSD 18M EE Animal 2	32386.03	19.23 ± 0.42	0.03
HDSD 18M EE Animal 3	11443.29	18.6 ± 0.62	0.04
HDSD 18M EE Animal 4	30412.63	25.06 ± 0.88	0.03
HDSD 18M EE Animal 5	32329.89	22.78 ± 0.35	0.03
Mean	27034.42	21.05 ± 0.5	0.03
Standard Error	3959.98	1.24 ± 0.12	
CV^2	0.107		
CE^2	0.001		
CE^2 / CV^2	0.01		
CVB^2	0.106		
CVB^2 (% of CV^2)	99.03%		
**HDSDHD / 18M / Enriched Environment**			
**Subjects**	**N**	**Thickness (μm)**	**CE (Scheaffer)**
HDSDHD 18M EE Animal 1	26718.43	20.56 ± 0.95	0.03

HDSDHD 18M EE Animal 2	16828.71	21.28 ± 0.22	0.04
HDSDHD 18M EE Animal 3	28586.49	21.13 ± 0.97	0.03
HDSDHD 18M EE Animal 4	8636.91	16.85 ± 1.02	0.04
HDSDHD 18M EE Animal 5	37781.14	24.98 ± 0.46	0.03
Mean	23710.34	20.96 ± 0.72	0.03
Standard Error	5026.82	1.29 ± 0.16	
CV^2	0.225		
CE^2	0.001		
CE^2 / CV^2	0.005		
CVB^2	0.224		
CVB^2 (% of CV^2)	99.5%		

$CVB^2 = CV^2 - CE^2$ (CV, coefficient of variation; CVB, biological coefficient of variation; CE, coefficient of error). N = number of astrocytes; Mean = mean numbers in each group; 6M and 18M indicate 6 months old and 18 months old, respectively.

Table S8. Estimated Unilateral Numbers of Astrocytes (N) With the Coefficient of Error (CE) for the Hilus of Dentate Gyrus of 6-, and 18-Month-Old Female Albino Swiss Mice Fed a Hard Diet (HD), Hard/Soft Diet (HD/SD) and Hard/Soft/Hard Diet (HD/SD/HD).

**HILUS – DENTATE GYRUS**			
**HD / 6M / Impoverished Environment**			
**Subjects**	**N**	**Thickness (µm)**	**CE (Scheaffer)**
HD 6M IE Animal 1	19735.97	25.99 ± 0.17	0.04
HD 6M IE Animal 2	24046.63	26.58 ± 0.02	0.04
HD 6M IE Animal 3	20681.74	25.74 ± 0.32	0.04
HD 6M IE Animal 4	12045.6	25.32 ± 0.17	0.05
HD 6M IE Animal 5	16625.83	25.46 ± 0.13	0.04
Mean	18627.15	25.82 ± 0.16	0.04

Standard Error	2026.62	0.22 ± 0.05	
CV^2	0.06		
CE^2	0.002		
CE^2 / CV^2	0.03		
CVB^2	0.06		
CVB^2 (% of CV^2)	97.14%		
**HDSD / 6M / Impoverished Environment**			
**Subjects**	**N**	**Thickness (μm)**	**CE (Scheaffer)**
HDSD 6M IE Animal 1	13997.31	25.68 ± 1.08	0.06
HDSD 6M IE Animal 2	14231.49	24.45 ± 0.74	0.05
HDSD 6M IE Animal 3	22969.97	25.41 ± 0.51	0.04
HDSD 6M IE Animal 4	15060.26	23.51 ± 0.33	0.05
HDSD 6M IE Animal 5	10736.2	22.39 ± 0.49	0.05
Mean	15399.05	24.29 ± 0.63	0.05
Standard Error	2031.06	0.61 ± 0.13	
CV^2	0.087		
CE^2	0.002		
CE^2 / CV^2	0.026		
CVB^2	0.085		
CVB^2 (% of CV^2)	97.39%		
**HDSDHD / 6M / Impoverished Environment**			
**Subjects**	**N**	**Thickness (μm)**	**CE (Scheaffer)**
HDSDHD 6M IE Animal 1	16826.91	22.4 ± 1.12	0.04
HDSDHD 6M IE Animal 2	17438.66	25.32 ± 0.13	0.05
HDSDHD 6M IE Animal 3	26649.09	25.67 ± 0.1	0.03
HDSDHD 6M IE Animal 4	12255.09	22.2 ± 0.37	0.05
HDSDHD 6M IE Animal 5	13760.23	22.54 ± 0.48	0.05
Mean	17385.99	23.63 ± 0.44	0.04
Standard Error	2505.92	0.77 ± 0.18	
CV^2	0.104		
CE^2	0.002		
CE^2 / CV^2	0.019		
CVB^2	0.102		
CVB^2 (% of CV^2)	98.13%		

HD / 18M / Impoverished Environment			
**Subjects**	**N**	**Thickness (µm)**	**CE (Scheaffer)**
HD 18M IE Animal 1	18123	24.97 ± 0.35	0.04
HD 18M IE Animal 2	13088.06	24.6 ± 0.39	0.05
HD 18M IE Animal 3	14561.14	25.41 ± 0.43	0.05
HD 18M IE Animal 4	15676.89	24.27 ± 0.95	0.05
HD 18M IE Animal 5	17948.06	24.93 ± 0.51	0.05
Mean	15879.43	24.83 ± 0.53	0.03
Standard Error	971.69	0.19 ± 0.11	
CV^2	0.019		
CE^2	0.002		
CE^2 / CV^2	0.122		
CVB^2	0.016		
CVB^2 (% of CV^2)	87.85%		
**HDSD / 18M / Impoverished Environment**			
**Subjects**	**N**	**Thickness (µm)**	**CE (Scheaffer)**
HDSD 18M IE Animal 1	13951.71	22.97 ± 0.71	0.05
HDSD 18M IE Animal 2	17.23	25.01 ± 0.74	0.05
HDSD 18M IE Animal 3	17454.34	24.22 ± 0.95	0.05
HDSD 18M IE Animal 4	6989.49	24.21 ± 1.05	0.06
HDSD 18M IE Animal 5	12233.31	26.34 ± 0.29	0.05
Mean	10129.22	24.55 ± 0.75	0.05
Standard Error	3039.79	0.55 ± 0.13	
CV^2	0.450		
CE^2	0.002		
CE^2 / CV^2	0.005		
CVB^2	0.448		
CVB^2 (% of CV^2)	99.46%		
**HDSDHD / 18M / Impoverished Environment**			
**Subjects**	**N**	**Thickness (µm)**	**CE (Scheaffer)**
HDSDHD 18M IE Animal 1	10299.26	26.46 ± 0.44	0.06
HDSDHD 18M IE Animal 2	13260.34	23.34 ± 0.66	0.05
HDSDHD 18M IE Animal 3	18426.34	21.75 ± 0.47	0.04
HDSDHD 18M IE Animal 4	10342.29	24.11 ± 0.37	0.05

HDSDHD 18M IE Animal 5	15353.14	22.69 ± 0.96	0.04
Mean	13536.27	23.67 ± 0.58	0.05
Standard Error	1548.71	0.8 ± 0.11	
CV^2	0.065		
CE^2	0.002		
CE^2 / CV^2	0.034		
CVB^2	0.063		
CVB^2 (% of CV^2)	96.55%		
**HD / 6M / Enriched Environment**			
**Subjects**	**N**	**Thickness (μm)**	**CE (Scheaffer)**
HD 6M EE Animal 1	16734.26	23.67 ± 0.59	0.04
HD 6M EE Animal 2	14331.69	21.53 ± 0.58	0.05
HD 6M EE Animal 3	12114.43	21.61 ± 1.06	0.05
HD 6M EE Animal 4	20569.97	22.71 ± 0.63	0.05
HD 6M EE Animal 5	18649.11	23.49 ± 0.31	0.05
Mean	16479.89	22.6 ± 0.63	0.05
Standard Error	1502.85	0.45 ± 0.12	
CV^2	0.042		
CE^2	0.002		
CE^2 / CV^2	0.054		
CVB^2	0.039		
CVB^2 (% of CV^2)	94.62%		
**HDSD / 6M / Enriched Environment**			
**Subjects**	**N**	**Thickness (μm)**	**CE (Scheaffer)**
HDSD 6M EE Animal 1	7829.31	19.13 ± 0.96	0.05
HDSD 6M EE Animal 2	20270.14	20.07 ± 0.66	0.04
HDSD 6M EE Animal 3	25596.26	20.94 ± 0.47	0.04
HDSD 6M EE Animal 4	18766.37	19.7 ± 0.59	0.04
HDSD 6M EE Animal 5	13943.66	19.81 ± 0.44	0.05
Mean	17281.15	19.93 ± 0.62	0.04
Standard Error	3006.30	0.3 ± 0.09	
CV^2	0.151		
CE^2	0.002		
CE^2 / CV^2	0.012		

CVB^2	0.15		
CVB^2 (% of CV^2)	98.84%		
**HDSDHD / 6M / Enriched Environment**			
**Subjects**	**N**	**Thickness (µm)**	**CE (Scheaffer)**
HDSDHD 6M EE Animal 1	17734.46	16.29 ± 0.87	0.04
HDSDHD 6M EE Animal 2	10518.51	17.37 ± 0.66	0.05
HDSDHD 6M EE Animal 3	9962.57	20.55 ± 0.71	0.05
HDSDHD 6M EE Animal 4	19193.14	21.01 ± 0.35	0.04
HDSDHD 6M EE Animal 5	16498.11	23.87 ± 0.91	0.05
Mean	14781.36	19.82 ± 0.7	0.05
Standard Error	1904.26	1.36 ± 0.1	
CV^2	0.083		
CE^2	0.002		
CE^2 / CV^2	0.026		
CVB^2	0.081		
CVB^2 (% of CV^2)	97.38%		
**HD / 18M / Enriched Environment**			
**Subjects**	**N**	**Thickness (µm)**	**CE (Scheaffer)**
HD 18M EE Animal 1	13646.14	18.98 ± 0.45	0.05
HD 18M EE Animal 2	21006.17	22.73 ± 1.27	0.04
HD 18M EE Animal 3	15297.86	21.45 ± 0.93	0.04
HD 18M EE Animal 4	16604.91	20.28 ± 0.32	0.04
HD 18M EE Animal 5	13901.83	23.57 ± 0.13	0.05
Mean	16091.38	21.4 ± 0.62	0.04
Standard Error	1338.32	0.82 ± 0.21	
CV^2	0.035		
CE^2	0.002		
CE^2 / CV^2	0.059		
CVB^2	0.033		
CVB^2 (% of CV^2)	94.14%		
**HDSD / 18M / Enriched Environment**			
**Subjects**	**N**	**Thickness (µm)**	**CE (Scheaffer)**
HDSD 18M EE Animal 1	19327.89	18.86 ± 0.41	0.05
HDSD 18M EE Animal 2	17348.83	19.16 ± 0.26	0.05

HDSD 18M EE Animal 3	7131.51	19.14 ± 0.72	0.05
HDSD 18M EE Animal 4	19113.77	24.3 ± 0.87	0.04
HDSD 18M EE Animal 5	18293.57	22.72 ± 0.46	0.04
Mean	16243.11	20.83 ± 0.54	0.04
Standard Error	2304.39	1.12 ± 0.11	
CV^2	0.101		
CE^2	0.002		
CE^2 / CV^2	0.019		
CVB^2	0.099		
CVB^2 (% of CV^2)	98.08%		
**HDSDHD / 18M / Enriched Environment**			
**Subjects**	**N**	**Thickness (μm)**	**CE (Scheaffer)**
HDSDHD 18M EE Animal 1	15587.14	20.02 ± 1.13	0.04
HDSDHD 18M EE Animal 2	10102.11	20.96 ± 0.47	0.05
HDSDHD 18M EE Animal 3	15152.49	21.52 ± 0.44	0.05
HDSDHD 18M EE Animal 4	4169.49	16.4 ± 0.52	0.06
HDSDHD 18M EE Animal 5	18137.31	23.52 ± 0.25	0.04
Mean	12629.71	20.48 ± 0.56	0.05
Standard Error	2484.04	1.17 ± 0.15	
CV^2	0.193		
CE^2	0.002		
CE^2 / CV^2	0.012		
CVB^2	0.191		
CVB^2 (% of CV^2)	98.8%		

$CVB^2 = CV^2 - CE^2$ (CV, coefficient of variation; CVB, biological coefficient of variation; CE, coefficient of error). N = number of astrocytes; Mean = mean numbers in each group; 6M and 18M indicate 6 months old and 18 months old, respectively.
